# Supplementary material for: Identification of significant chromatin contacts from HiChIP data by FitHiChIP
Source: Nat Commun. 2019 Sep 17;10:4221. doi: 10.1038/s41467-019-11950-y (PMC6748947; doi:10.1038/s41467-019-11950-y)
Supplement: Supplementary file 1 — Supplementary Information [file 41467_2019_11950_MOESM1_ESM.pdf]

Supplementary information for Identification of significant chromatin contacts from HiChIP data by FitHiChIP

Bhattacharyya et. al.

August 13, 2019

Supplementary Figures

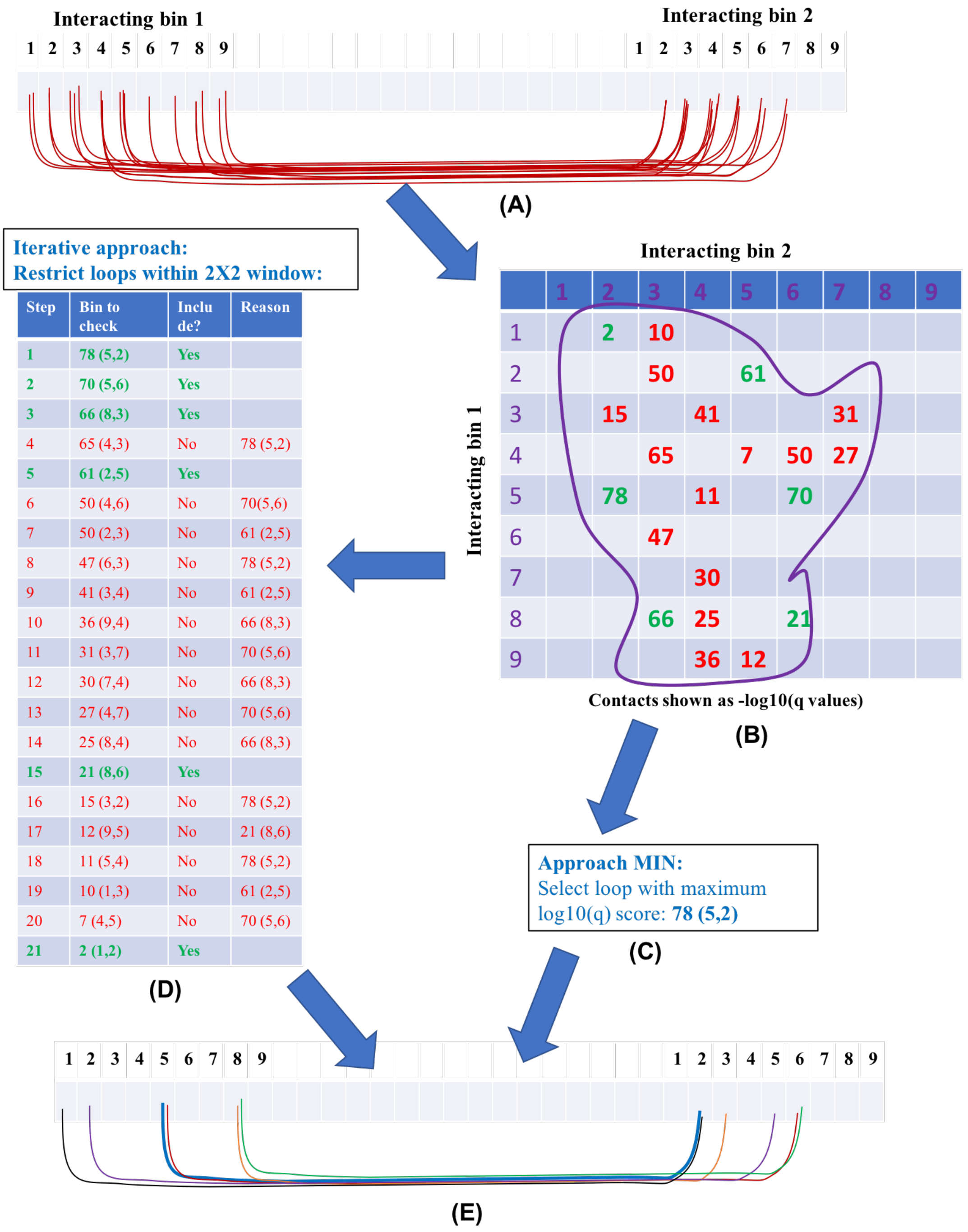

Supplementary Figure 1: **Schematic of the iterative execution of *merging filter* on adjacent loops of a connected component identified using an 8-connectivity based algorithm.** **(A)**: An example of a pair of regions with multiple adjacent loop calls. **(B)**: Matrix representation of loop calls within the connected component using their minus log10(q-value) scores (any other score such as contact/PET counts would also work). **(C)**: Schematic of a trivial approach (**MIN**), which chooses per component, the loop with minimum q-value (maximum significance). **(D)**: Step by step execution of our merging filter, namely the **ITERATIVE** approach, which iteratively includes a loop l with current maximum minus log10(q-value) in the final set of loops F, provided l does not belong within a window W of $k \times k$ (k=2 in terms of bins) neighborhood of any loop present in F. Otherwise the loop is discarded (colored in red), and the conflicting loops in F are also indicated. **(E)**: The set of loops returned by the **ITERATIVE** approach, by definition includes the most significant loop returned by the **MIN** approach (highlighted in blue). FitHiChIP uses the **ITERATIVE** approach with W=2 $\times$ 2.

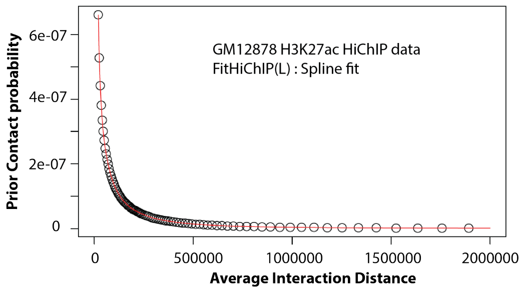

(a) Spline fit for peak-to-all background: using loops whose at least one end is a *peak bin*.

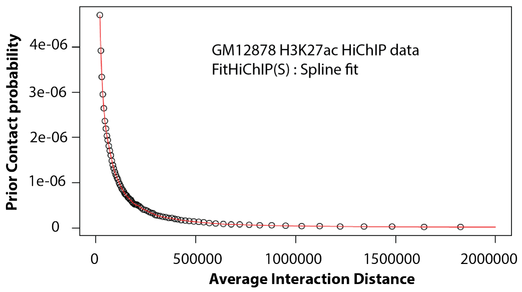

(b) Spline fit for peak-to-peak background: using loops whose both ends overlap with *peak bins*.

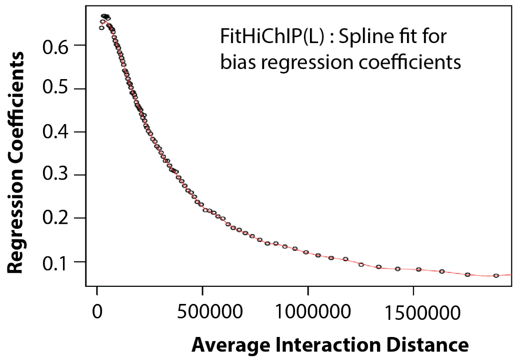

(c) Spline fit f_{β_1} of the regression coefficients β_1^j (j: bin index) for the bias values B_1^j (of one end of interacting loci within an equal occupancy bin) for peak-to-all background

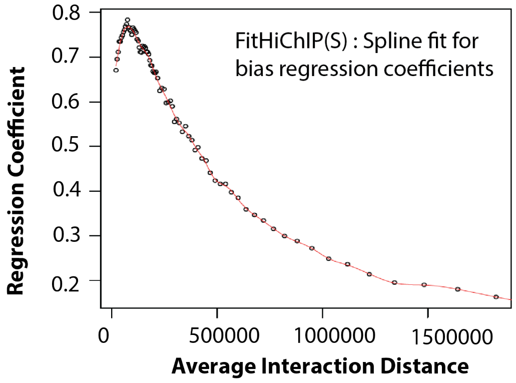

(d) Spline fit f_{β_1} of the regression coefficients β_1^j (j: bin index) for the bias values B_1^j (of one end of interacting loci within an equal occupancy bin) for peak-to-peak background

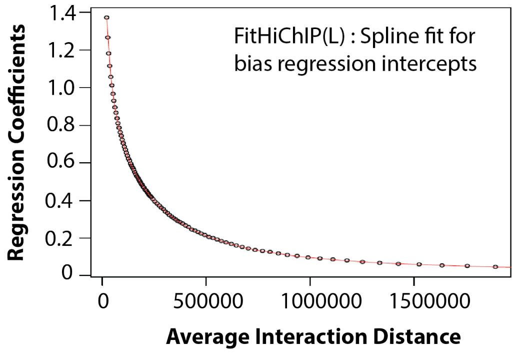

(e) Spline fit f_{β_0} of the intercept terms β_0^j (j: bin index) for peak-to-all background

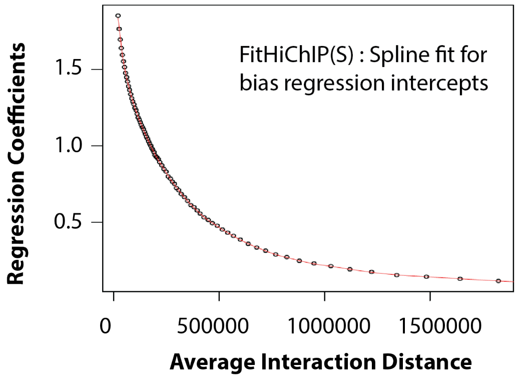

(f) Spline fit f_{β_0} of the intercept terms β_0^j(j: bin index) for peak-to-peak background

Supplementary Figure 2: **Stringent background model results in higher parameter estimates for spline fit and for regression coefficients (a)-(b)**: Average contact probability and spline fit for loose (**a**) and stringent (**b**) background of FitHiChIP, when applied on GM12878 H3K27ac HiChIP data [20]. **(c)-(d)**: Spline fit of bias regression coefficients for individual equal occupancy bins, corresponding to the loose (**c**) and stringent (**d**) backgrounds. **(e)-(f)**: Spline fit of intercept coefficients for individual equal occupancy bins, corresponding to the loose (**e**) and stringent (**f**) backgrounds.

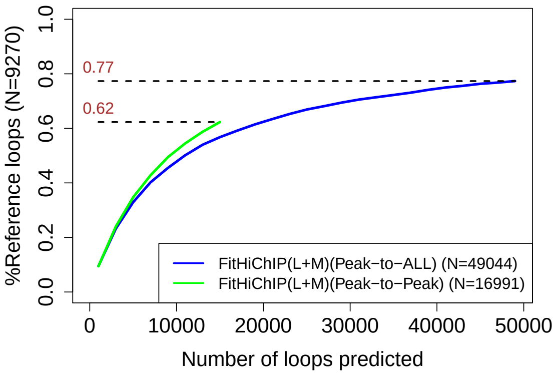

(a) **Dataset:** GM12878 cohesin HiChIP. **Reference:** HiCCUPS loops from GM12878 Hi-C.

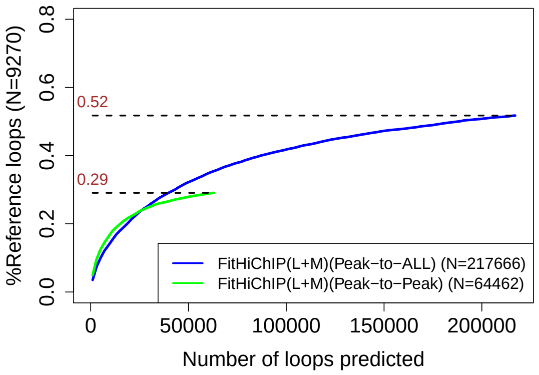

(b) **Dataset:** GM12878 H3K27ac HiChIP. **Reference:** HiCCUPS loops from GM12878 Hi-C.

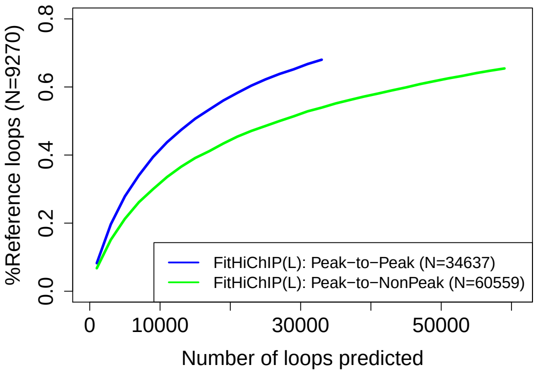

(c) **Dataset:** GM12878 cohesin HiChIP. **Reference:** HiCCUPS loops from GM12878 Hi-C.

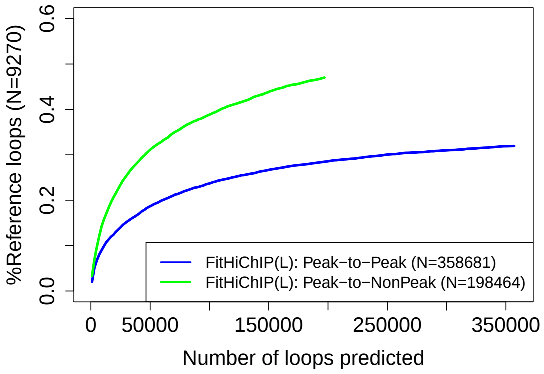

(d) **Dataset:** GM12878 H3K27ac HiChIP. **Reference:** HiCCUPS loops from GM12878 Hi-C.

Supplementary Figure 3: **Peak-to-all foreground recovers more HiCCUPS Hi-C loops than peak-to-peak: (a)-(b):** FitHiChIP(L+M) with peak-to-all foreground (i.e., the set of loops considered for significance assignment), as used for all results in the current study, recovers a higher fraction of HiCCUPS Hi-C loops [23] compared to using peak-to-peak foreground from both GM12878 cohesin and GM12878 H3K27ac HiChIP data. **(c-d)**: Comparison of peak-to-peak and peak-to-nonpeak FitHiChIP loops in recovering reference *in situ* Hi-C loops from either cohesin or H3K27ac HiChIP data. A significant fraction of recovery is contributed by peak-to-nonpeak loops in H3K27ac data. The symbol 'N' indicates total number of loops for the corresponding method.

Recovery of *in situ* Hi-C HiCCUPS loops

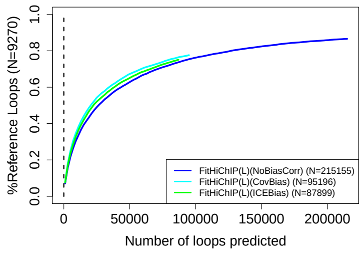

(a) **Dataset:** GM12878 cohesin HiChIP. **Reference:** HiCCUPS loops from GM12878 Hi-C.

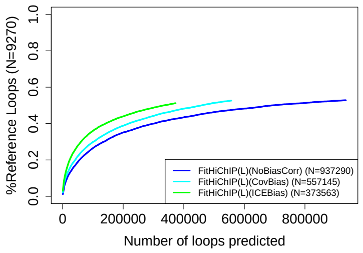

(b) **Dataset:** GM12878 H3K27ac HiChIP. **Reference:** HiCCUPS loops from GM12878 Hi-C.

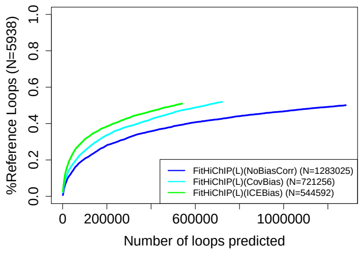

(c) **Dataset:** K562 H3K27ac HiChIP. **Reference:** HiCCUPS loops from K562 Hi-C.

Recovery of HiChIP HiCCUPS loops

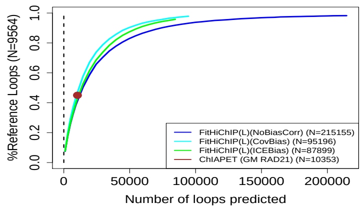

(d) **Dataset:** GM12878 cohesin HiChIP. **Reference:** HiCCUPS loops from GM12878 cohesin HiChIP.

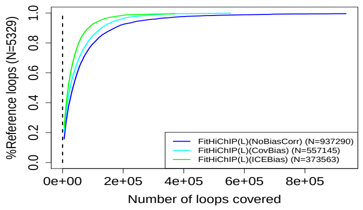

(e) **Dataset:** GM12878 H3K27ac HiChIP. **Reference:** HiCCUPS loops from GM12878 H3K27ac HiChIP.

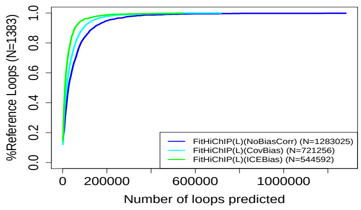

(f) **Dataset:** K562 H3K27ac HiChIP. **Reference:** HiCCUPS loops from K562 H3K27ac HiChIP.

Recovery of ChIA-PET loops

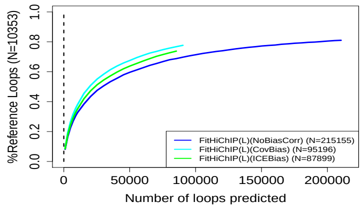

(g) **Dataset:** GM12878 cohesin HiChIP. **Reference:** ChIA-PET loops from GM12878 RAD21.

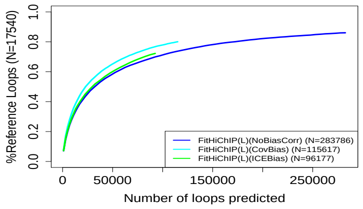

(h) **Dataset:** mES cohesin HiChIP. **Reference:** ChIA-PET loops from mES Smc1a.

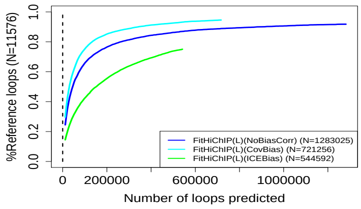

(i) **Dataset:** K562 H3K27ac HiChIP. **Reference:** ChIA-PET loops from K562 Pol-II.

Supplementary Figure 4: **FitHiChIP with either coverage or ICE bias regression shows better recovery of reference loops compared to not using any bias correction.**

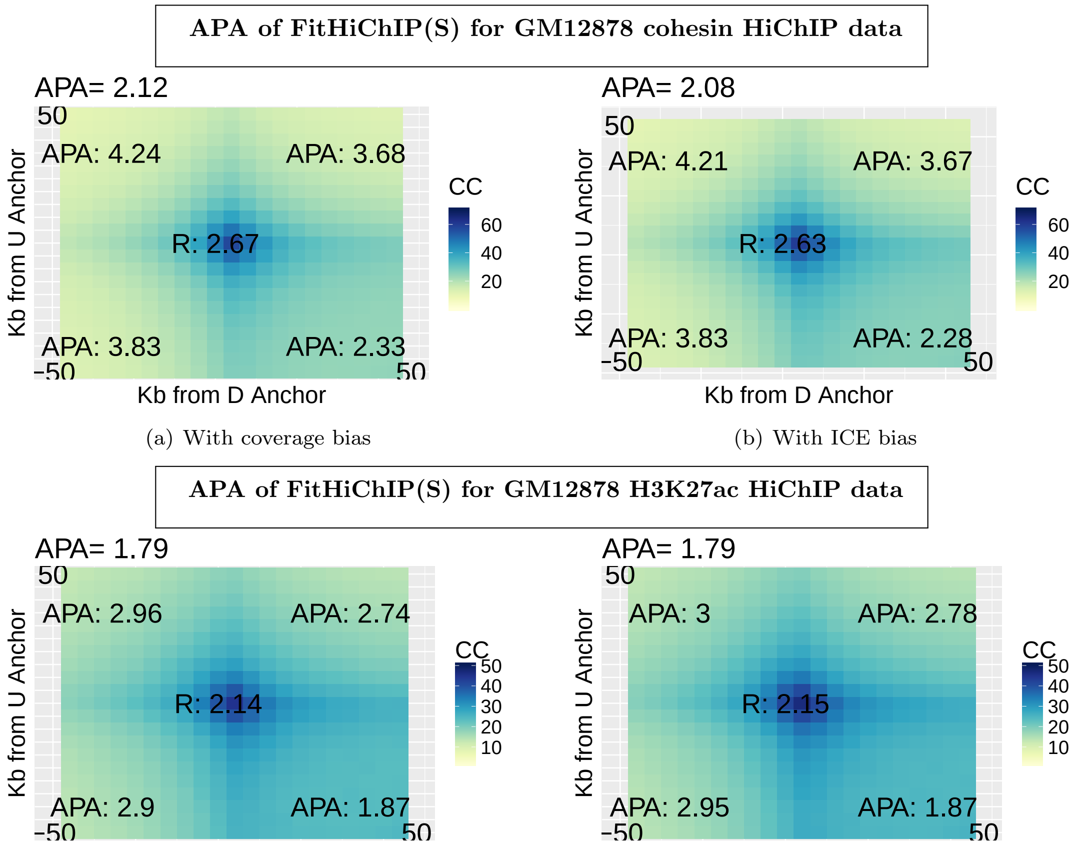

(a) With coverage bias (b) With ICE bias

(c) With coverage bias (d) With ICE bias

Supplementary Figure 5: **FitHiChIP(S) loops with either coverage or ICE bias exhibit similar APA scores for cohesin and H3K27ac HiChIP datasets.** We use the top 5441 loop calls for (a-b) and top 2629 loop calls for (c-d) to stay consistent with Fig. 2 (main manuscript) and Supplementary Figure 21 below.

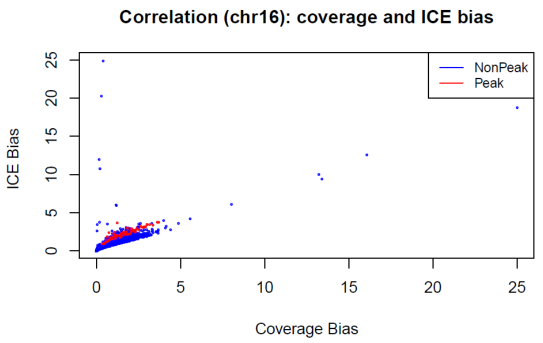

(a) GM12878 cohesin HiChIP

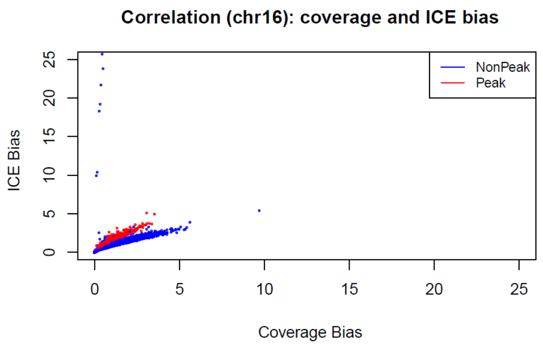

(b) mES cohesin HiChIP

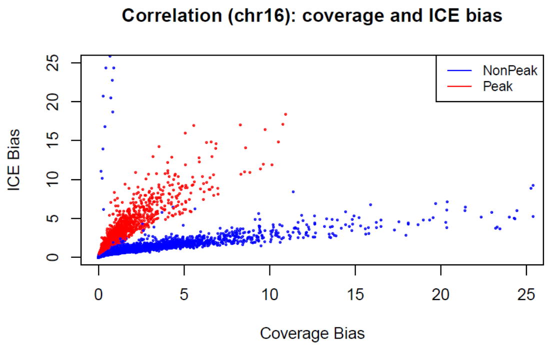

(c) GM12878 H3K27ac HiChIP

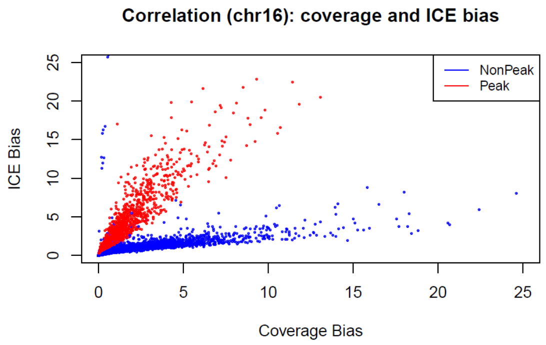

(d) K562 H3K27ac HiChIP

Supplementary Figure 6: **Comparison between coverage and ICE bias values for an example chromosome.** Coverage and ICE bias values (for chromosome 16) of peak and non-peak bins are shown for four different HiChIP datasets. These values show the presence of several artifacts, where non-peak regions with very low coverage (x nearly zero) end up having very high ICE bias values (y values over 5 or 10).

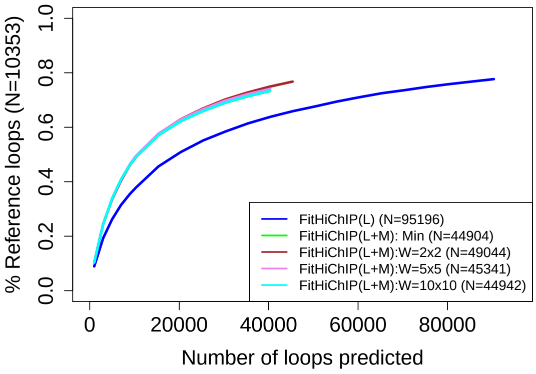

(a) **Dataset:** GM12878 cohesin HiChIP. **Reference:** GM12878 RAD21 ChIA-PET.

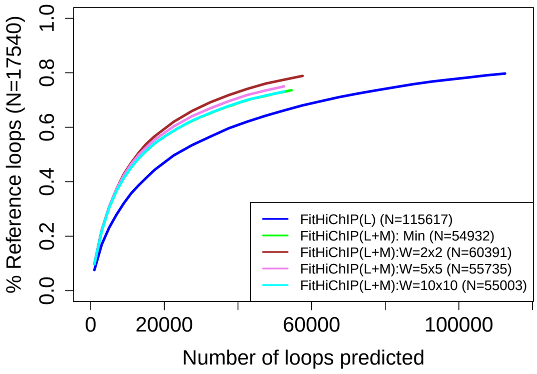

(b) **Dataset:** mES cohesin HiChIP. **Reference:** mES Smc1 ChIA-PET.

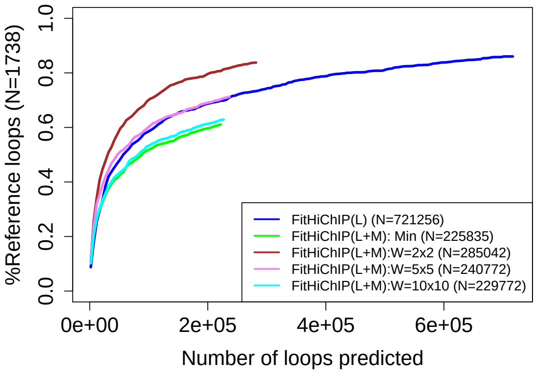

(c) **Dataset:** K562 H3K27ac HiChIP. **Reference:** K562 H3K27ac ChIA-PET.

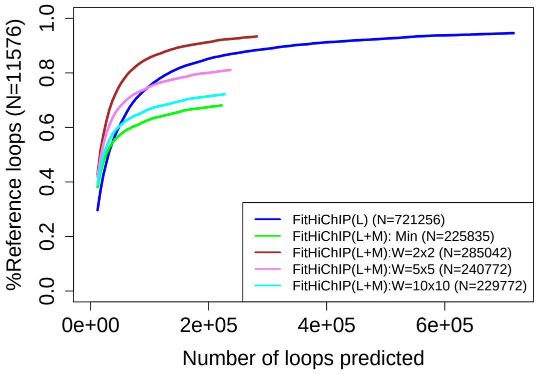

(d) **Dataset:** K562 H3K27ac HiChIP. **Reference:** K562 Pol-II ChIA-PET.

Supplementary Figure 7: **Testing different settings of merging filter for their recovery performance of reference ChIA-PET loops.** The *MIN* approach as well as three different values for window size (W) in terms of bins for the *ITERATIVE* approach (as described in Supplementary Figure 1) are tested against each other and no merging filter with respect to their recovery of reference ChIA-PET loops. The *ITERATIVE* approach with $W = 2 \times 2$ (brown lines) performs better and, hence, is used as default settings for our merging filter. All results use FitHiChIP(L) with coverage bias regression, and reference ChIP-seq peaks.

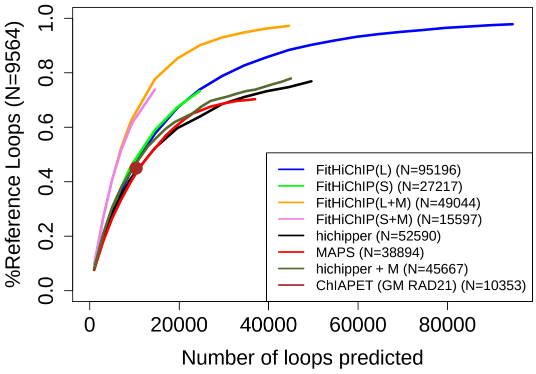

(a) **Dataset:** GM12878 cohesin HiChIP. **Reference:** HiCCUPS loops from GM12878 cohesin HiChIP.

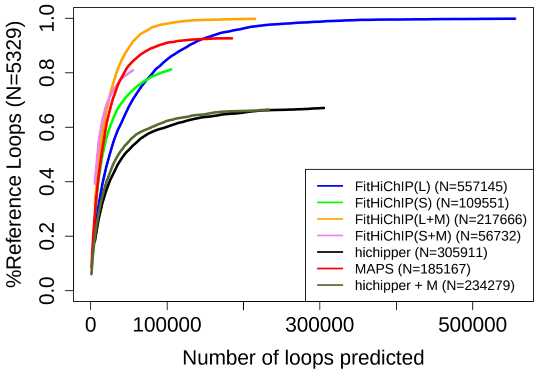

(b) **Dataset:** GM12878 H3K27ac HiChIP. **Reference:** HiCCUPS loops from GM12878 H3K27ac HiChIP.

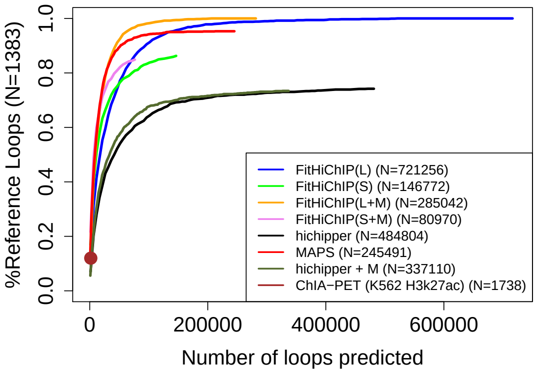

(c) **Dataset:** K562 H3K27ac HiChIP. **Reference:** HiCCUPS loops from K562 H3K27ac HiChIP.

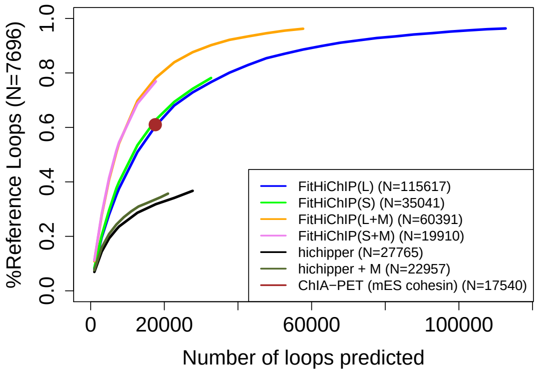

(d) **Dataset:** mES cohesin HiChIP. **Reference:** HiCCUPS loops from mES cohesin HiChIP.

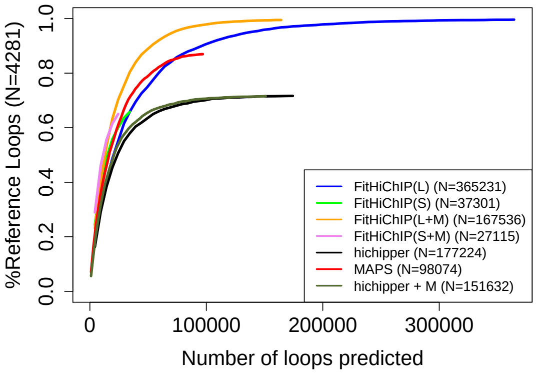

(e) **Dataset:** CD4 naïve H3K27ac HiChIP. **Reference:** HiCCUPS loops from CD4 naïve H3K27ac HiChIP.

Supplementary Figure 8: **Recovery of HiCCUPS loops on HiChIP data using reference ChIP-seq peaks.** FitHiChIP shows better recovery of reference HiCCUPS loops (computed from HiChIP data) compared to hichipper and MAPS when reference ChIP-seq peaks are used by all methods. Here hichipper + M indicates our proposed *merging filter* is applied to hichipper loops. FitHiChIP is run with coverage bias regression. Only the loops within distance range 20 Kb - 2 Mb are considered. The symbol N denotes the number of significant loops generated by the corresponding method. MAPS produced error during processing of mES cohesin HiCHIP data, so no loops were generated. Brown dots indicate the fraction of overlap (single value) between the reference HiCCUPS loops and the ChIA-PET loops for the corresponding dataset. Note that for this analysis, we use HiCCUPS as a reference method rather than for comparison purposes as HiCCUPS reports a stringent set of loops with high specificity.

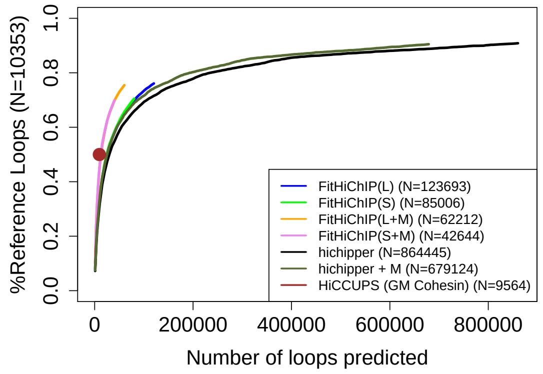

(a) **Dataset**: GM12878 cohesin HiChIP. **Reference**: GM12878 RAD21 ChIA-PET.

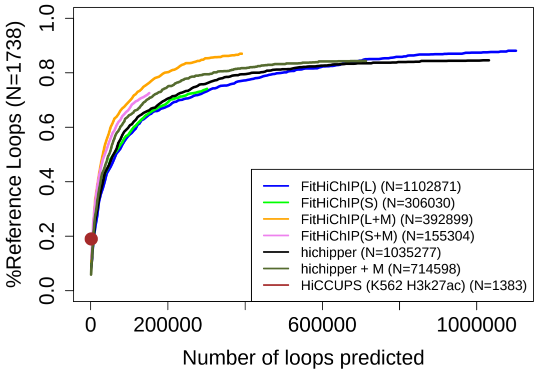

(b) **Dataset**: K562 H3K27ac HiChIP. **Reference**: K562 H3K27ac ChIA-PET.

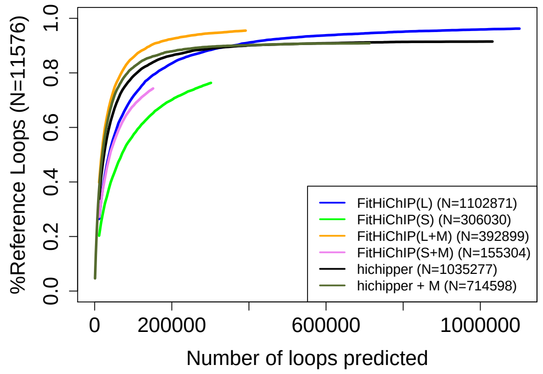

(c) **Dataset**: K562 H3K27ac HiChIP. **Reference**: K562 Pol-II ChIA-PET.

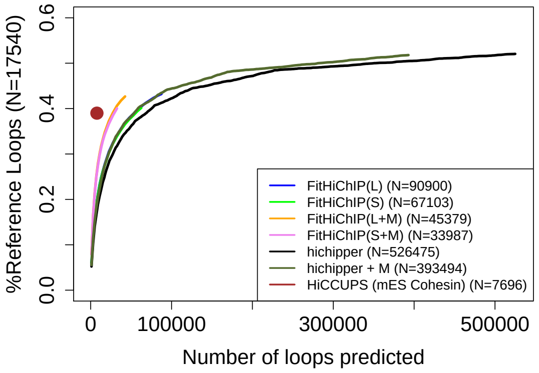

(d) **Dataset**: mES cohesin HiChIP. **Reference**: mES smc1 ChIA-PET.

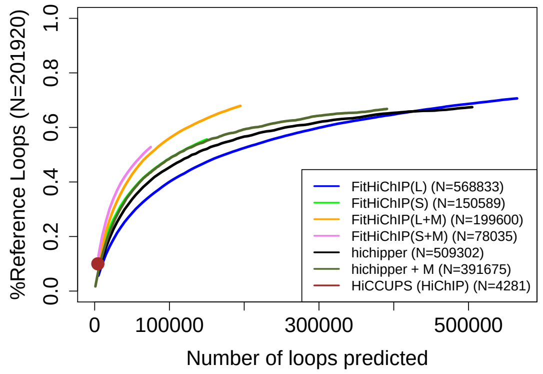

(e) **Dataset**: CD4 naïve H3K27ac HiChIP. **Reference**: CD4 naïve PCHiC.

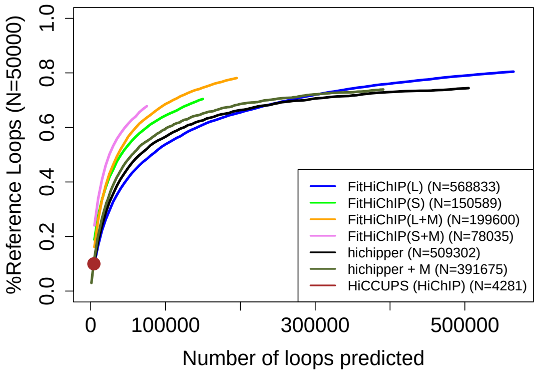

(f) **Dataset**: CD4 naïve H3K27ac HiChIP. **Reference**: Top 50K CD4 naïve PCHiC loops.

Supplementary Figure 9: **Recovery of ChIA-PET and promoter capture Hi-C loops using peaks inferred from HiChIP data itself.** Similar to using ChIP-seq peaks (Supplementary Figure 15), FitHiChIP shows better recovery performance compared to hichipper for both ChIA-PET and PCHiC loops when HiChIP peaks are used. Note that for this case, hichipper calls many times more loops compared to both FitHiChIP and also to hichipper calls when reference ChIP-seq peaks are used (Supplementary Figure 15). All settings and notations are similar to Supplementary Figure 8.

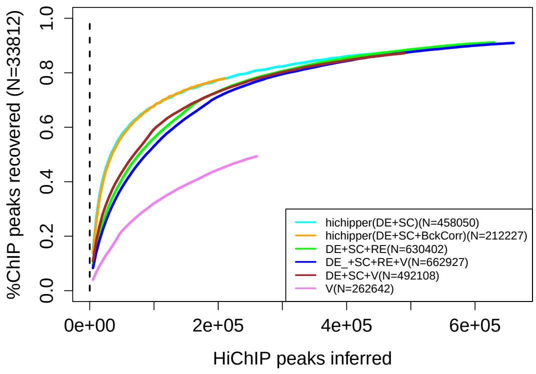

(a) **Dataset:** GM12878 cohesin HiChIP peaks. **Reference:** GM12878 SMC3 ChIP-seq peaks.

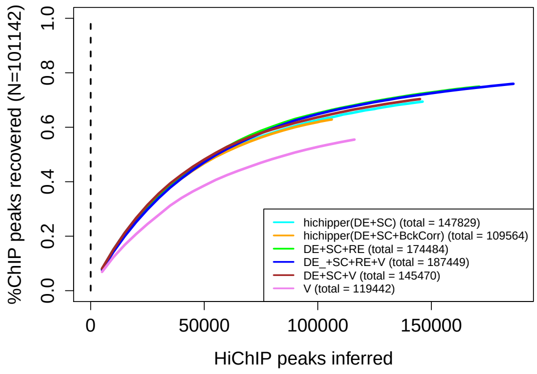

(b) **Dataset:** GM12878 H3K27ac HiChIP peaks. **Reference:** GM12878 H3K27ac ChIP-seq peaks.

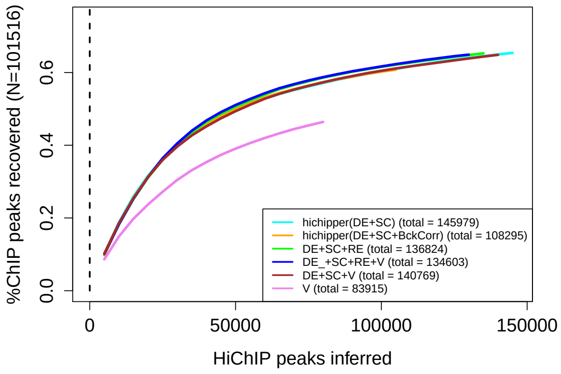

(c) **Dataset:** K562 H3K27ac HiChIP peaks. **Reference:** K562 H3K27ac ChIP-seq peaks.

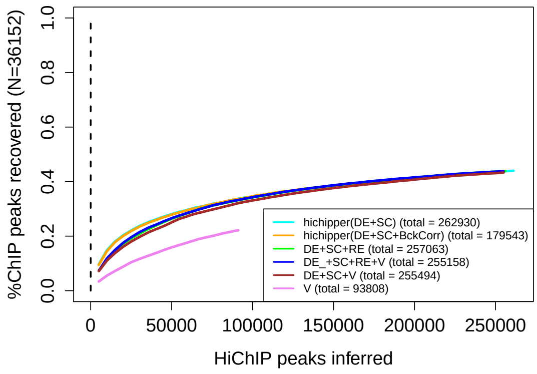

(d) **Dataset:** mES cohesin HiChIP peaks. **Reference:** mES SMC3 ChIP-seq peaks.

Supplementary Figure 10: **Overlap between reference ChIP-seq peaks and HiChIP inferred peaks.** Peaks are inferred from HiChIP data using different groups of reads (generated by HiC-pro pipeline) and with or without the RE site bias correction proposed by hichipper. The orange line indicates peaks inferred by hichipper with its custom background correction. Rest of the peaks are derived by HiChIP reads without hichipper specific background correction. Peaks are compared to the corresponding set of reference ChIP-seq peak calls. The recovery performance is very similar for all settings except using only valid (V) reads, which performs worse. For GM12878 cohesin data, hichipper with background correction shows a slight improvement in specificity. As suggested in [14], we extend peaks by 1kb slack to compute overlap.

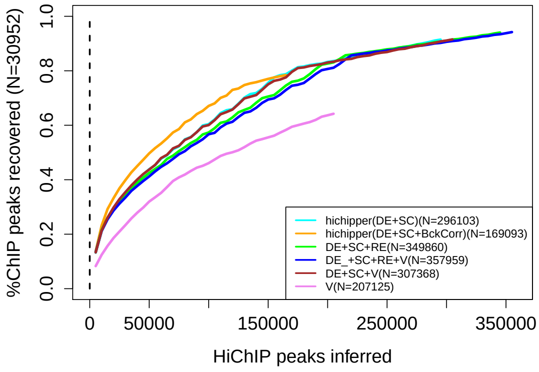

(a) **Dataset:** GM12878 cohesin HiChIP peaks. **Reference:** GM12878 SMC3 ChIP-seq peaks.

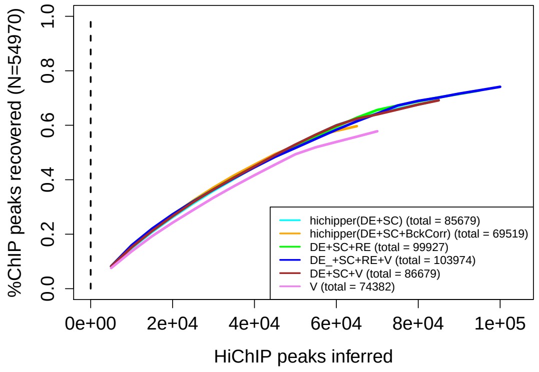

(b) **Dataset:** GM12878 H3K27ac HiChIP peaks. **Reference:** GM12878 H3K27ac ChIP-seq peaks.

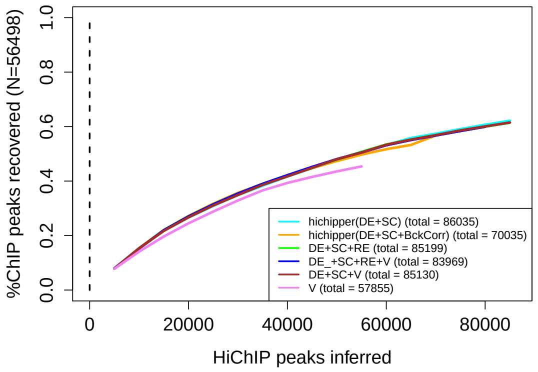

(c) **Dataset:** K562 H3K27ac HiChIP peaks. **Reference:** K562 H3K27ac ChIP-seq peaks.

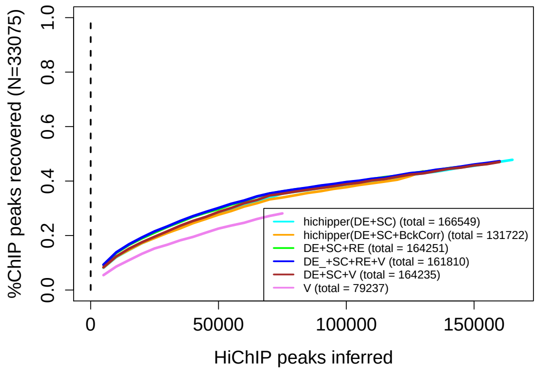

(d) **Dataset:** mES cohesin HiChIP peaks. **Reference:** mES SMC3 ChIP-seq peaks.

Supplementary Figure 11: **Overlap between reference ChIP-seq peaks and HiChIP inferred peaks at the level of 5kb bins.** Similar overlap analysis to Supplementary Figure 10 when peak calls from HiChIP and ChIP-seq are both assigned to 5kb bins before computing overlaps on binary peak/non-peak labels.

Recovery of *in situ* Hi-C HiCCUPS loops

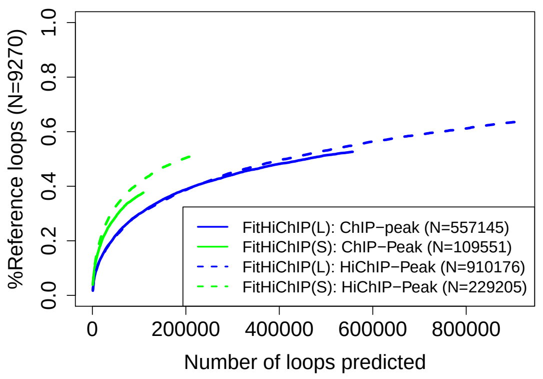

(a) **Dataset:** GM12878 H3K27ac HiChIP. **Reference:** HiCCUPS loops from GM12878 Hi-C.

Recovery of HiChIP HiCCUPS loops

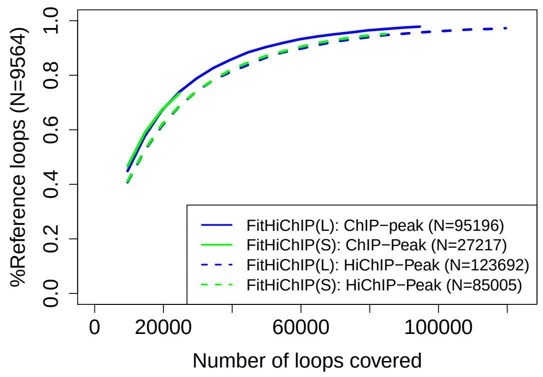

(b) **Dataset:** GM12878 cohesin HiChIP. **Reference:** HiCCUPS loops from GM12878 cohesin HiChIP.

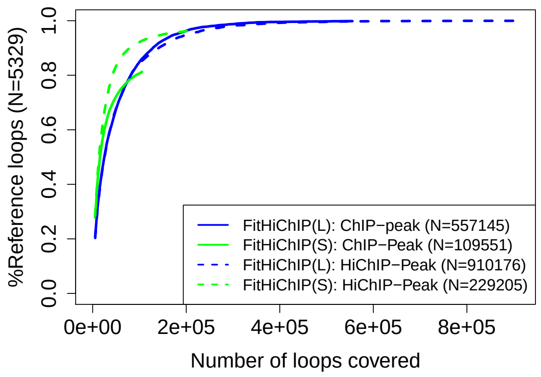

(c) **Dataset:** GM12878 H3K27ac HiChIP. **Reference:** HiCCUPS loops from GM12878 H3K27ac HiChIP.

Recovery of ChIA-PET loops

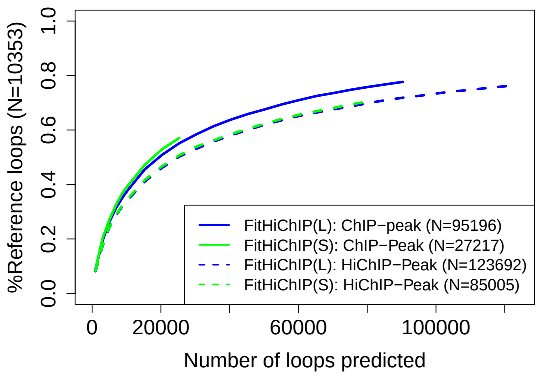

(d) **Dataset**: GM12878 cohesin HiChIP. **Reference**: GM12878 RAD21 ChIA-PET.

Supplementary Figure 12: **Comparison of FitHiChIP performance when it uses peaks either from a reference ChIP-seq data or inferred from HiChIP data.** The performance is measured by either using HiCCUPS loop calls from GM12878 *in situ* Hi-C data or from the HiChIP dataset, on which FitHiChIP is run. A similar analysis is done also using GM12878 RAD21 ChIA-PET loops as reference [9].

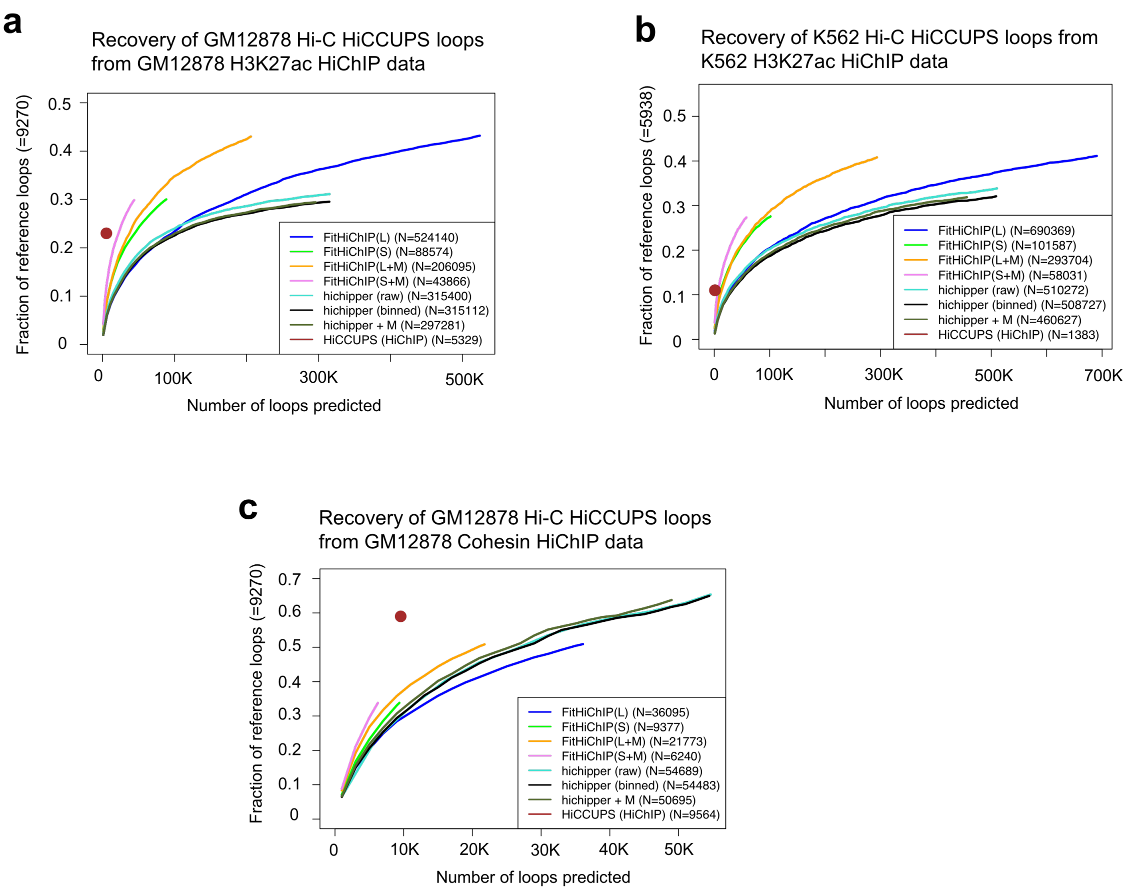

Supplementary Figure 13: **FitHiChIP executed with 2.5kb binning show higher recovery of reference HiCCUPS loops from *in-situ* Hi-C datasets, compared to hichipper.** Brown dots in these plots correspond to the output of HiChIP HiCCUPS loops having either 5kb or 10kb resolution (same as those employed for recovery plots of 5Kb FitHiChIP or hichipper loops in main paper, Fig. 2). Cyan line shows performance of raw hichipper loops (PET count ≥ 2, without any binning), and the black line shows performance of binned (2.5 Kb) hichipper loops. The method hichipper + M (dark green line) applies our proposed *merging filter* routine on the binned hichipper loops. We note that resolution of reference loops are not changed; only the resolution of FitHiChIP and hichipper loops are changed for comparison.

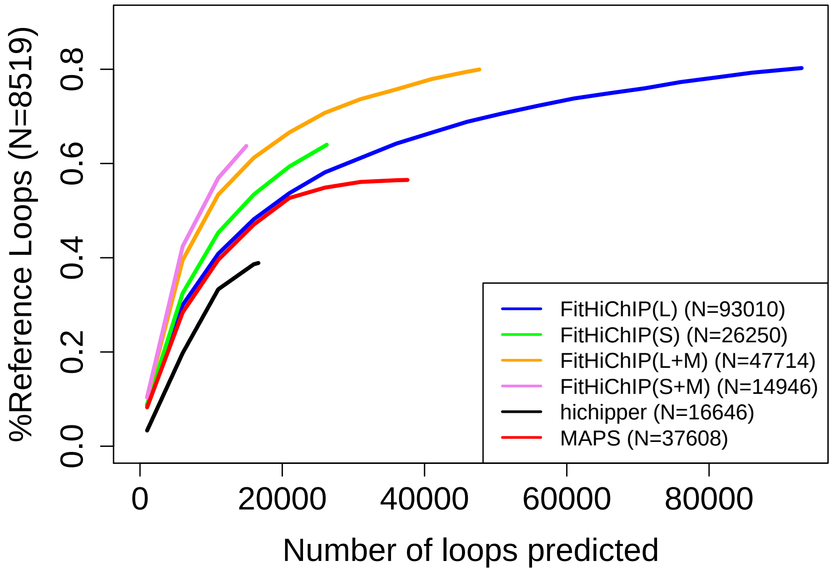

(a) **Dataset**: GM12878 cohesin HiChIP. **Reference**: GM12878 Hi-C HiCCUPS loops.

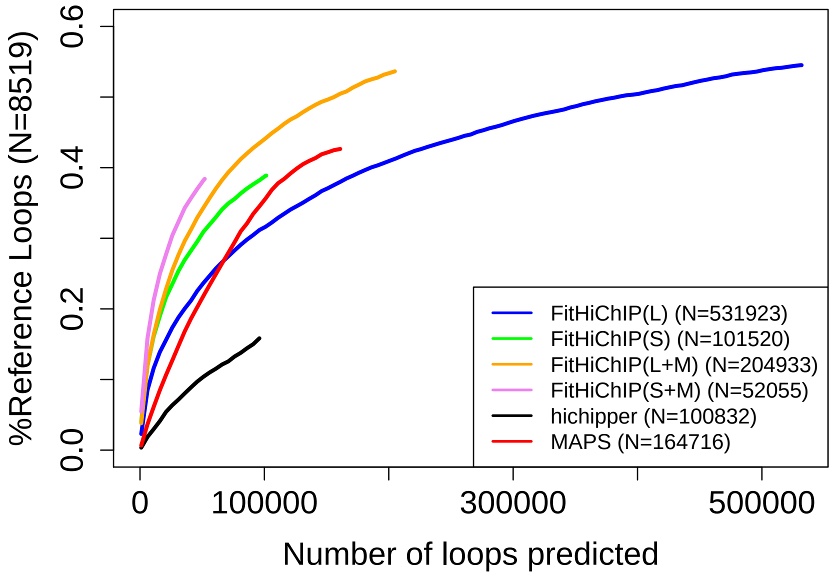

(b) **Dataset**: GM12878 H3K27ac HiChIP. **Reference**: GM12878 Hi-C HiCCUPS loops.

Supplementary Figure 14: **Comparison of FitHiChIP, hichipper and MAPS in recovering GM12878 *in-situ* Hi-C HiCCUPS loops when hichipper and MAPS loops from the supplementary data of MAPS (provided in [13]) are used. a**: Using loop calls from GM12878 cohesin HiChIP data. **b**: Using loop calls from GM12878 H3K27ac HiChIP data. Since 1Mb upper bound is used by MAPS for these loop calls, FitHiChIP loop calls are subsetted to the same distance range (20kb, 1Mb). Only autosomal chromosomes are used as described by MAPS.

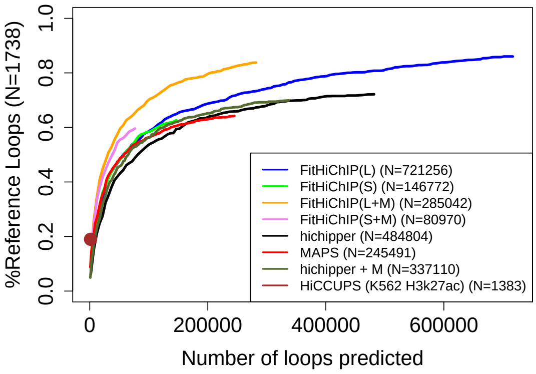

(a) **Dataset:** K562 H3K27ac HiChIP. **Reference:** K562 H3K27ac ChIA-PET.

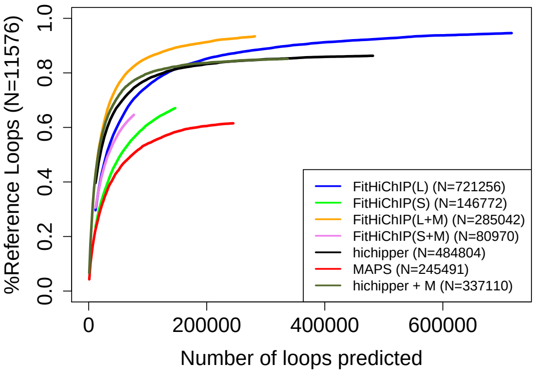

(b) **Dataset:** K562 H3K27ac HiChIP. **Reference:** K562 Pol-II ChIA-PET.

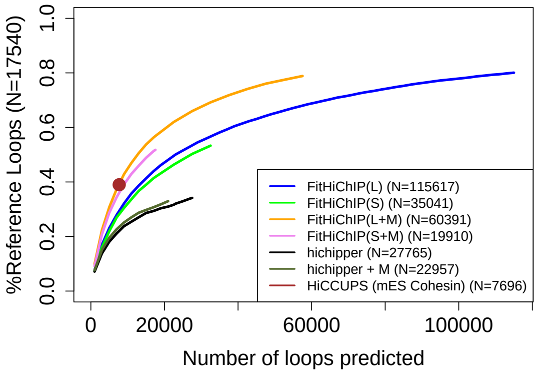

(c) **Dataset:** mES cohesin HiChIP. **Reference:** mES Smc1 ChIA-PET.

Supplementary Figure 15: **Recovery of ChIA-PET loops using reference ChIP-seq peaks.** FitHiChIP shows better recovery of reference ChIA-PET loops compared to hichipper and MAPS when reference ChIP-seq peaks are used by both methods. Performance comparison follows similar settings as in Supplementary Figure 8. MAPS produced error during processing of mES cohesin HiCHIP data, so no loops were generated.

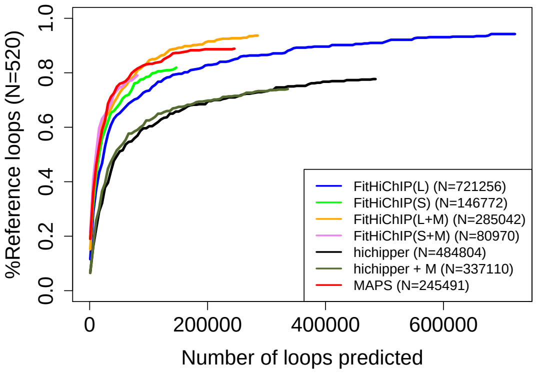

(a) **Dataset:** K562 H3K27ac HiChIP. **Reference:** Common loops between K562 H3K27ac ChIA-PET and K562 Hi-C HiCCUPS loops.

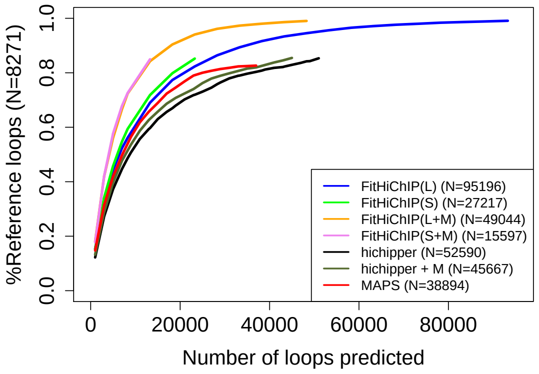

(b) **Dataset:** GM12878 cohesin HiChIP. **Reference:** Common loops between GM12878 RAD21 ChIA-PET and GM12878 cohesin HiChIP HiCCUPS calls.

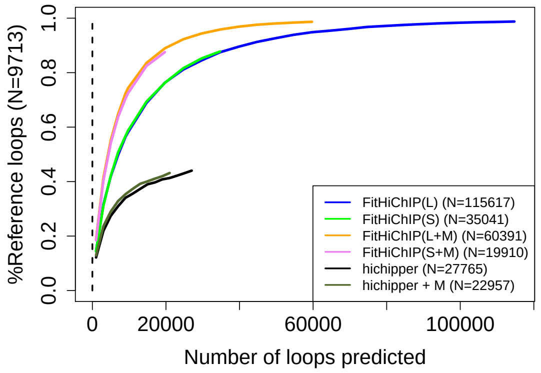

(c) **Dataset:** mES cohesin HiChIP. **Reference:** Common loops between mES Smc1 ChIA-PET and mES cohesin HiChIP HiCCUPS calls.

Supplementary Figure 16: **Recovery of loops common to matching ChIA-PET and HiCCUPS on HiChIP / Hi-C data using reference ChIP-seq peaks.** FitHiChIP shows better recovery compared to hichipper and MAPS. Reference ChIP-seq peaks are used by both methods. **(a)**: Common loops between K562 H3K27ac ChIA-PET loops [9] and K562 *in-situ* Hi-C HiCCUPS loops [23] are used as the reference. Common loops are derived by applying 5 Kb slack for loop overlap. Settings for the recovery plots are similar to Figure 8. **(b)**: Common loops between GM12878 Cohesin HiChIP HiCCUPS loops [19] and GM12878 RAD21 ChIA-PET loops [9] are used as reference. **(c)**: Common loops between mES Cohesin HiChIP HiCCUPS loops [19] and mES Smc1 ChIA-PET loops [5] are used as reference. MAPS produced errors in processing mES Cohesin HiChIP data, thereby could not generate any loops.

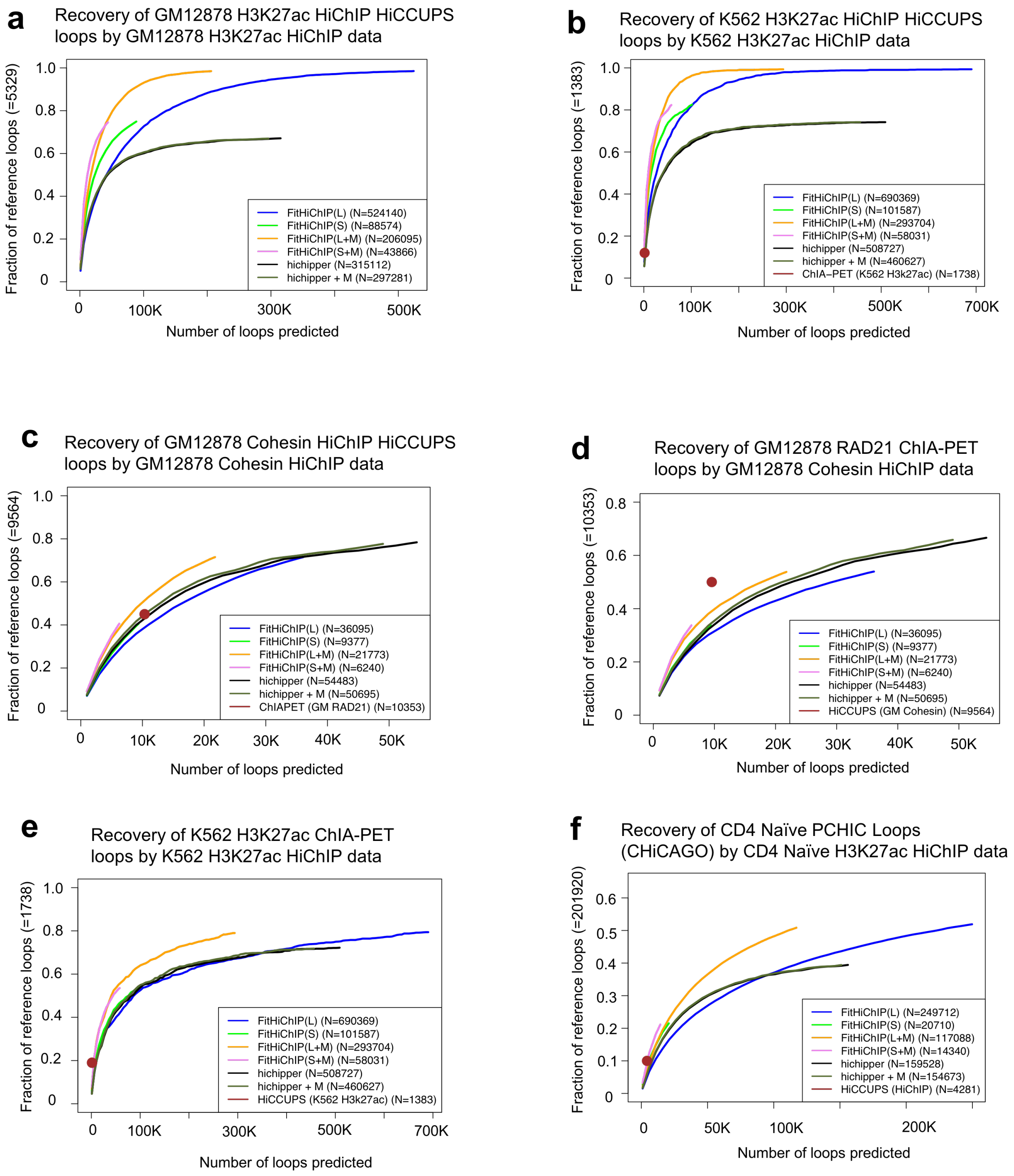

Supplementary Figure 17: **FitHiChIP executed with 2.5kb binning show higher recovery of reference ChIA-PET, HiCCUPS and promoter capture Hi-C loops, compared to hichipper.** **(a)-(c)**: Recovery of HiChIP HiCCUPS loops. **(d)-(e)**: Recovery of reference ChIA-PET loops. **(f)**: Recovery of CD4 naïve promoter capture Hi-C loops computed using ChICAGO. Brown dots in these plots correspond to the output of either HiChIP HiCCUPS loops or ChIA-PET loops having either 5 or 10kb resolution (same as those employed for recovery plots of 5kb FitHiChIP or hichipper loops). We note that resolution of reference loops or the HiChIP HiCCUPS or ChIA-PET loops are not changed; only the resolution of FitHiChIP and hichipper loops are changed for comparison. Here hichipper loops (shown in black line) are binned with respect to 2.5kb. Raw hichipper loops (without any binning) show similar recovery as the binned loops (as shown in Supplementary Figure 13), thus are not shown separately.

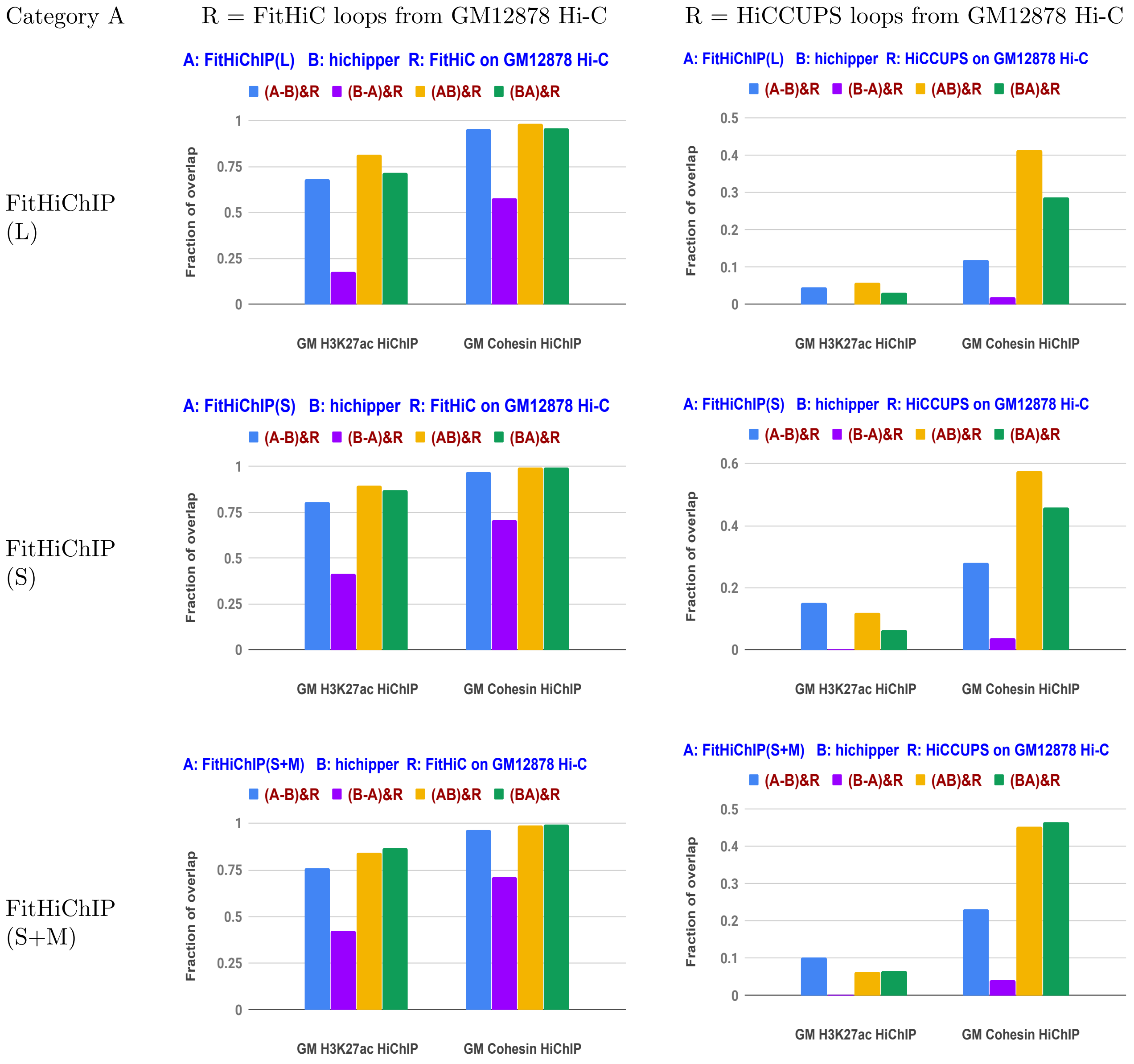

Supplementary Figure 18: **Intersection of overlapping and exclusive loops between FitHiChIP (rows indicate different settings) and hichipper, with a reference set of Hi-C loops either from FitHiC (first column) or from HiCCUPS (second column).** FitHiChIP and hichipper are applied on GM12878 H3K27ac [20] and GM12878 cohesin [19] HiChIP datasets. Loop overlap is computed using 5kb slack. *Notations*: A-B: Loops exclusive to A (not in B). AB: Loops common to A and B in the coordinates of set A. (A-B)&R: Loops in (A-B) which overlap with the reference set R.

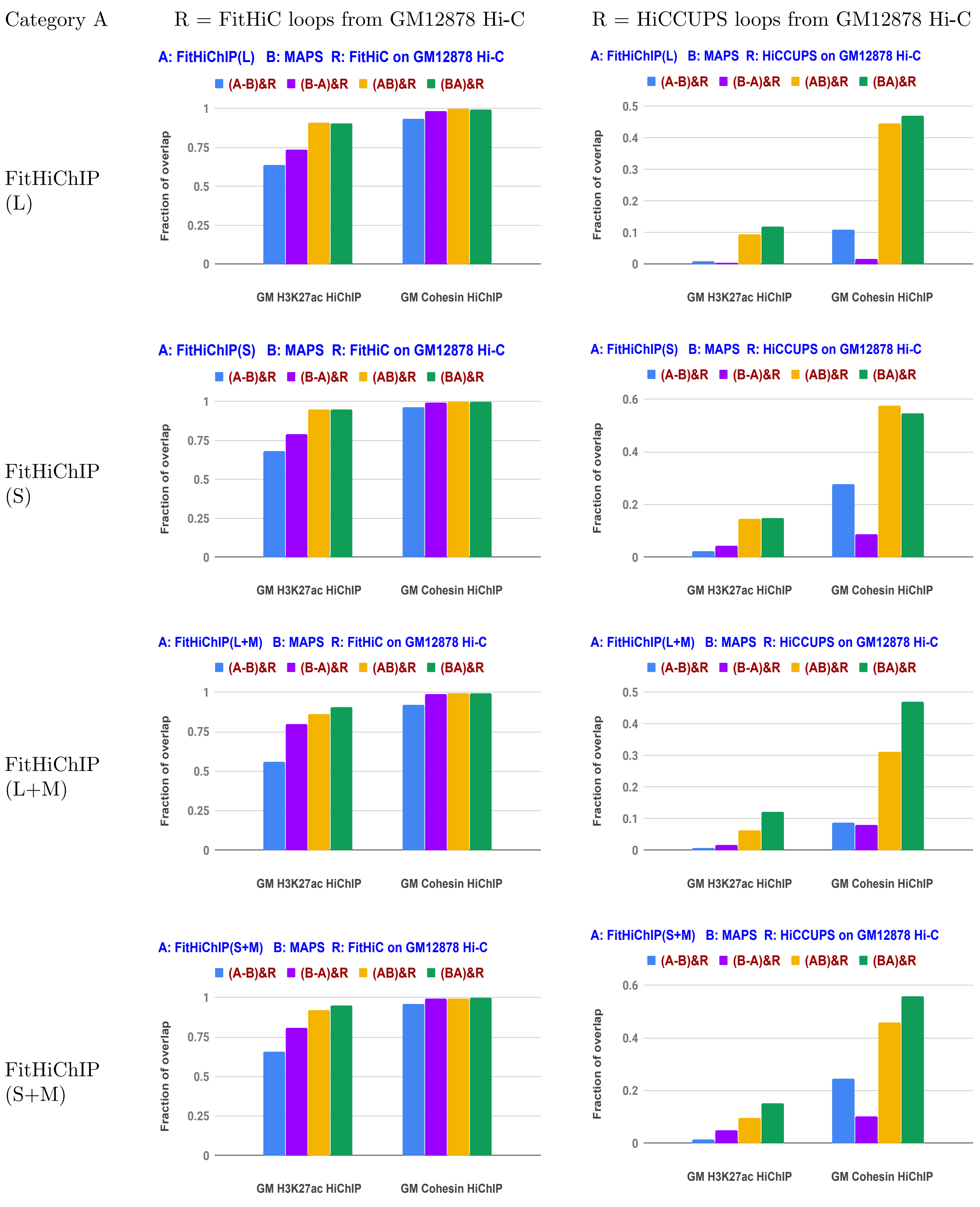

Supplementary Figure 19: **Intersection of overlapping and exclusive loops between FitHiChIP (rows indicate different settings) and MAPS, with a reference set of Hi-C loops either from FitHiC (first column) or from HiCCUPS (second column) of GM12878 *in situ* Hi-C data.** FitHiChIP and MAPS are applied on GM12878 H3K27ac [20] and GM12878 cohesin [19] HiChIP datasets. Loop overlap is computed using 5kb slack. *Notations*: A-B: Loops exclusive to A (not in B). AB: Loops common to A and B in the coordinates of set A. (A-B)&R: Loops in (A-B) which overlap with the reference set R.

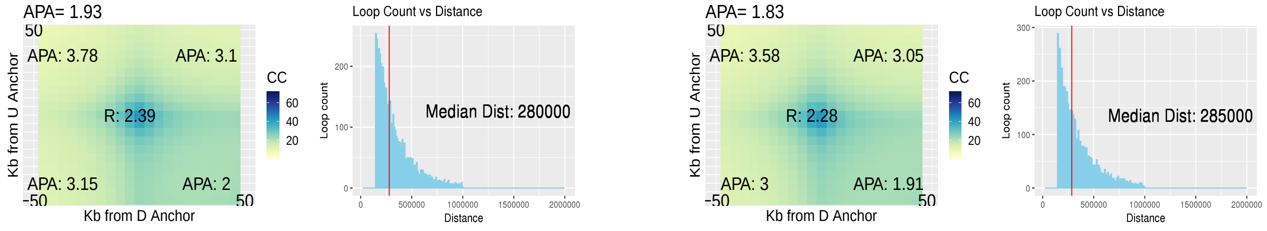

(a) HiCCUPS (5108 loops) [19] (b) Distance distribution (c) ChIA-PET (5441 loops) [9] (d) Distance distribution

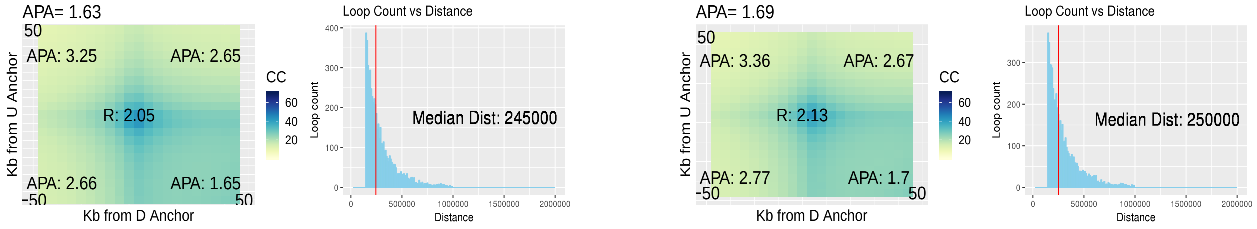

(e) hichipper (top 5441 loops) (f) Distance distribution (g) hichipper + M (top 5441 loops) (h) Distance distribution

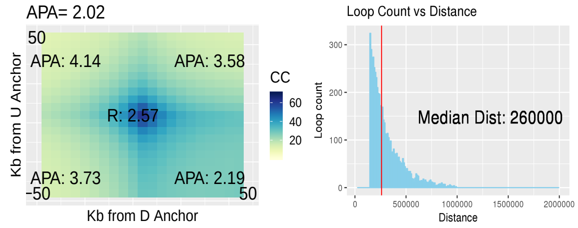

(i) MAPS (top 5441 loops) (j) Distance vs Loops

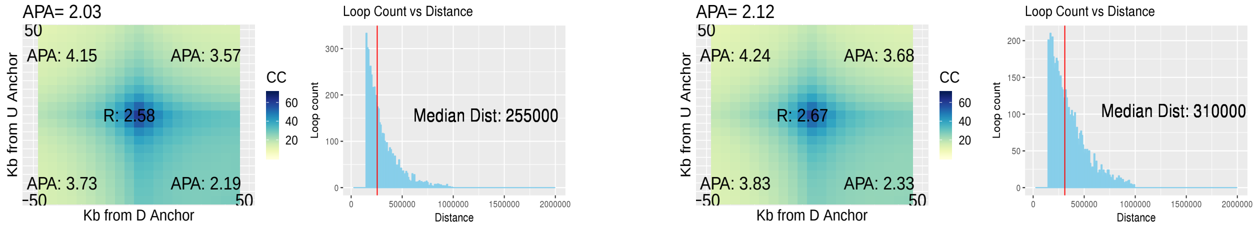

(k) FitHiChIP(L) (top 5441 loops) (l) Distance distribution (m) FitHiChIP(S) (top 5441 loops) (n) Distance distribution

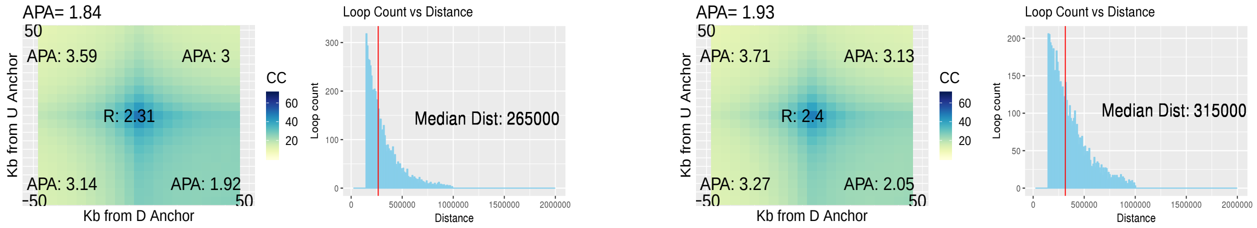

(o) FitHiChIP(L+M) (top 5441 loops) (p) Distance distribution (q) FitHiChIP(S+M) (top 5441 loops) (r) Distance distribution

Supplementary Figure 20: **Comparison of APA scores between FitHiChIP, hichipper and MAPS (top 5441 loops each) with the reference HiCCUPS (5108 loops) and ChIA-PET (5441 loops) interactions corresponding to the GM12878 cohesin data.** Loops within a distance of 150kb - 1 Mb are only considered (as suggested in [22]). For FitHiChIP and MAPS, top k loops are selected by higher statistical significance (lower q-values) whereas hichipper loops are selected by higher PET counts. APA scores displayed on top of each figure are computed with respect to 15-30kb downstream from the upstream interacting bin, and 15-30kb upstream from the downstream interacting bin (defined in [22]). In addition, APA scores for each corner region (10kb offset, as defined in [23]) are also shown. APA scores are computed using the Hi-C contact counts of GM12878 Hi-C data [23]. Also shown are the distance distributions for each loop set that are analyzed for APA scores plots.

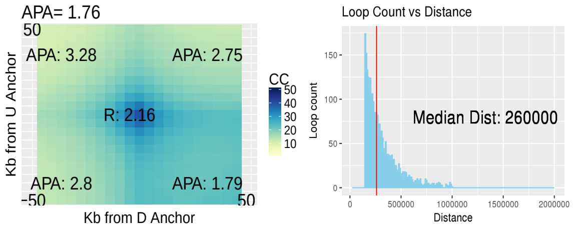

(a) HiCCUPS (2629 loops) [20] (b) Distance distribution

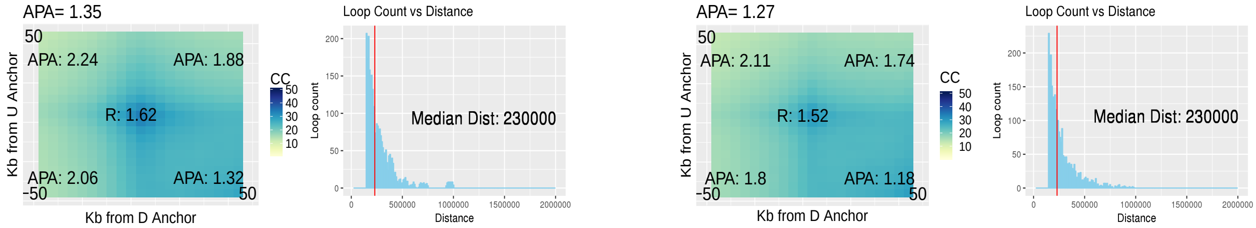

(c) hichipper (top 2629 loops) (d) Distance distribution (e) hichipper + M (top 2629 loops) (f) Distance distribution

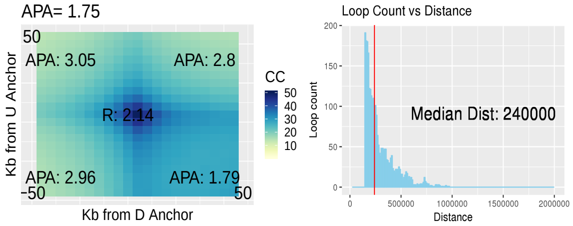

(g) MAPS (top 2629 loops) (h) Distance vs Loops

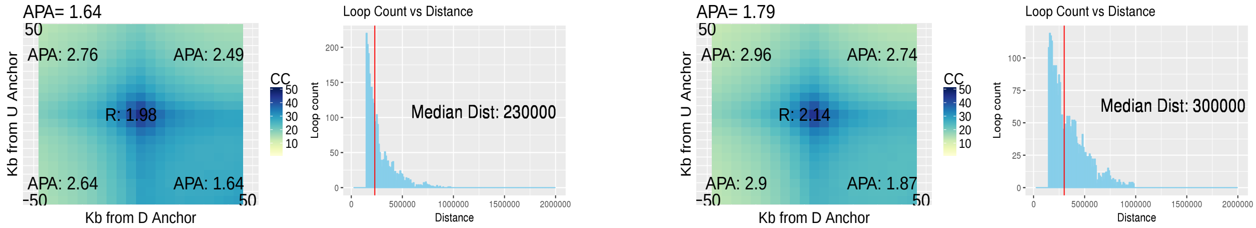

(i) FitHiChIP(L) (top 2629 loops) (j) Distance distribution (k) FitHiChIP(S) (top 2629 loops) (l) Distance distribution

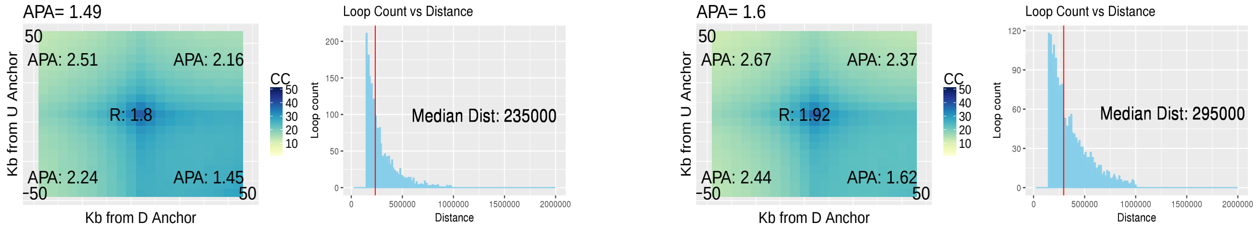

(m) FitHiChIP(L+M) (top 2629 loops) (n) Distance distribution (o) FitHiChIP(S+M) (top 2629 loops) (p) Distance distribution

Supplementary Figure 21: **Comparison of APA scores between FitHiChIP, hichipper and MAPS (top 2629 loops each) with the reference HiCCUPS (2629 loops) interactions, corresponding to the GM12878 H3K27ac HiChIP data [20].** APA scores are computed using similar settings mentioned in Supplementary Figure 20.

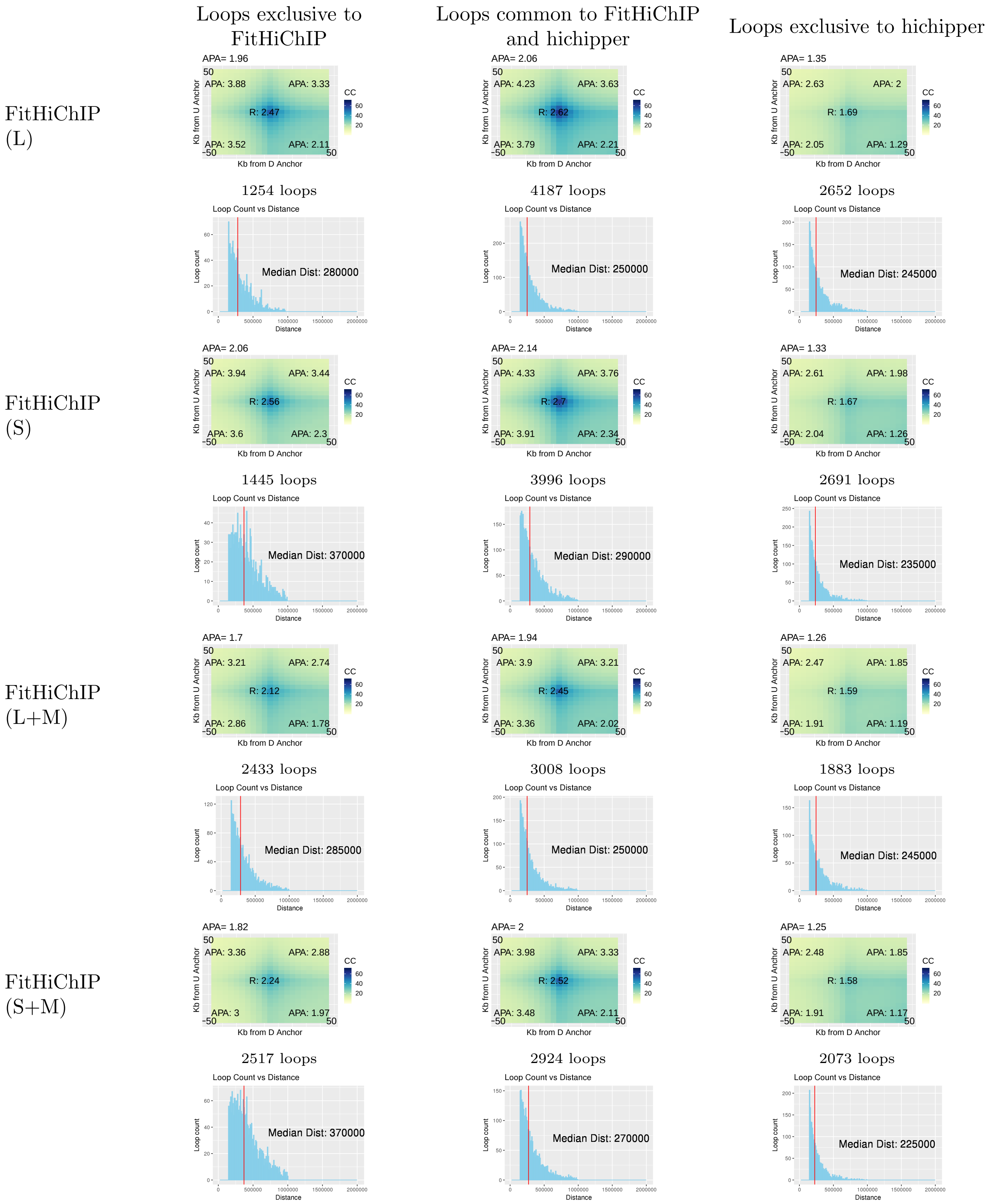

Supplementary Figure 22: **APA of overlapping and exclusive loops between FitHiChIP and hichipper for GM12878 cohesin HiChIP data [19].** Top-k loops of FitHiChIP (k=5441 within 150kb - 1Mb distance range, as mentioned in Fig. 20) report ∼30% to 40% exclusive interactions compared to hichipper loops. Exclusive loops for FitHiChIP (different settings) show higher APA scores compared to the exclusive hichipper loops, in spite of having higher median loop distance.

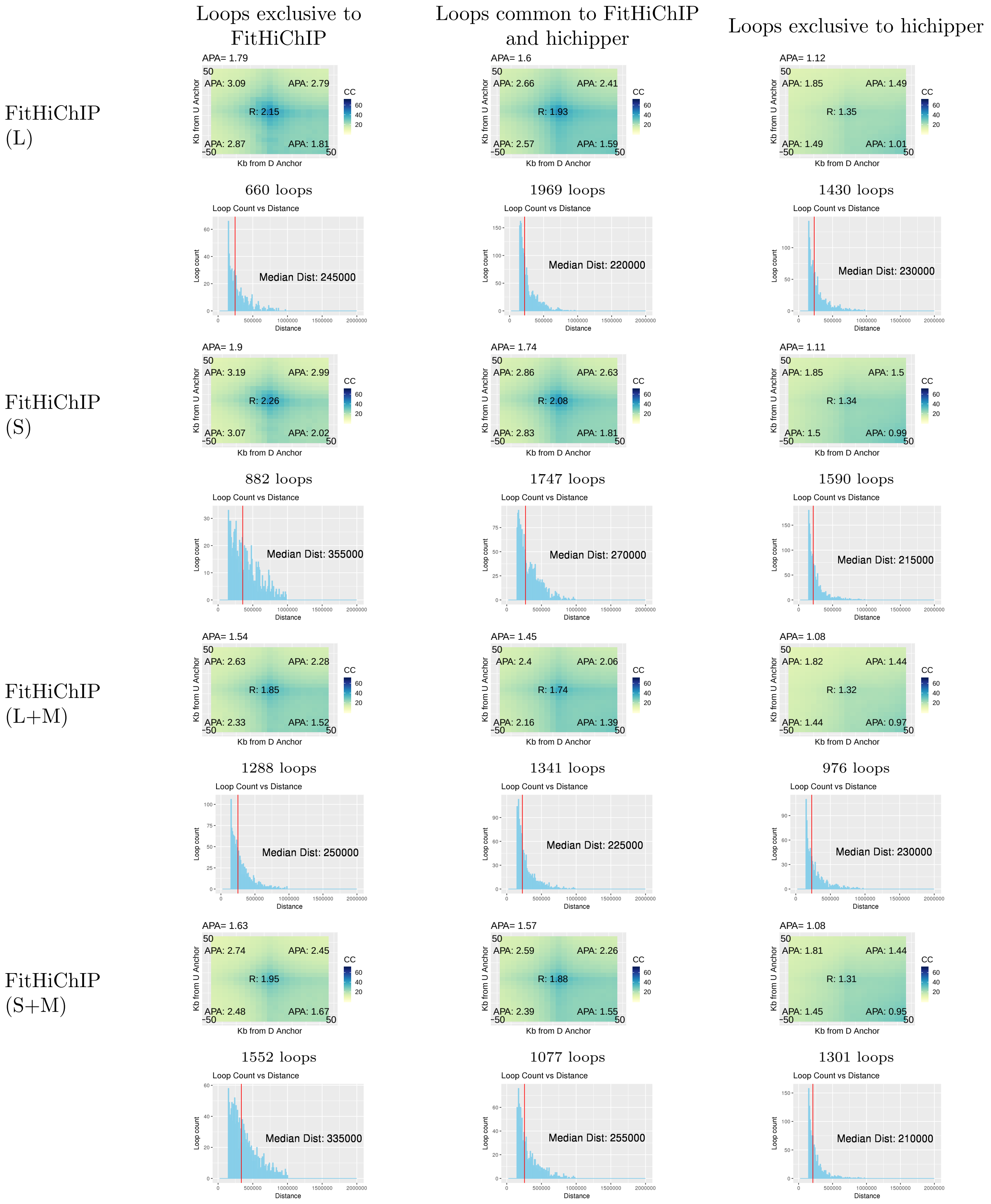

Supplementary Figure 23: **APA of overlapping and exclusive loops between FitHiChIP and hichipper for GM12878 H3K27ac HiChIP data [20].** Top-k loops of FitHiChIP (k=2629) contain ∼30% - 60% exclusive interactions compared to hichipper loops. Exclusive loops for FitHiChIP (different settings) show higher APA scores compared to the exclusive hichipper loops, in spite of having higher median loop distance.

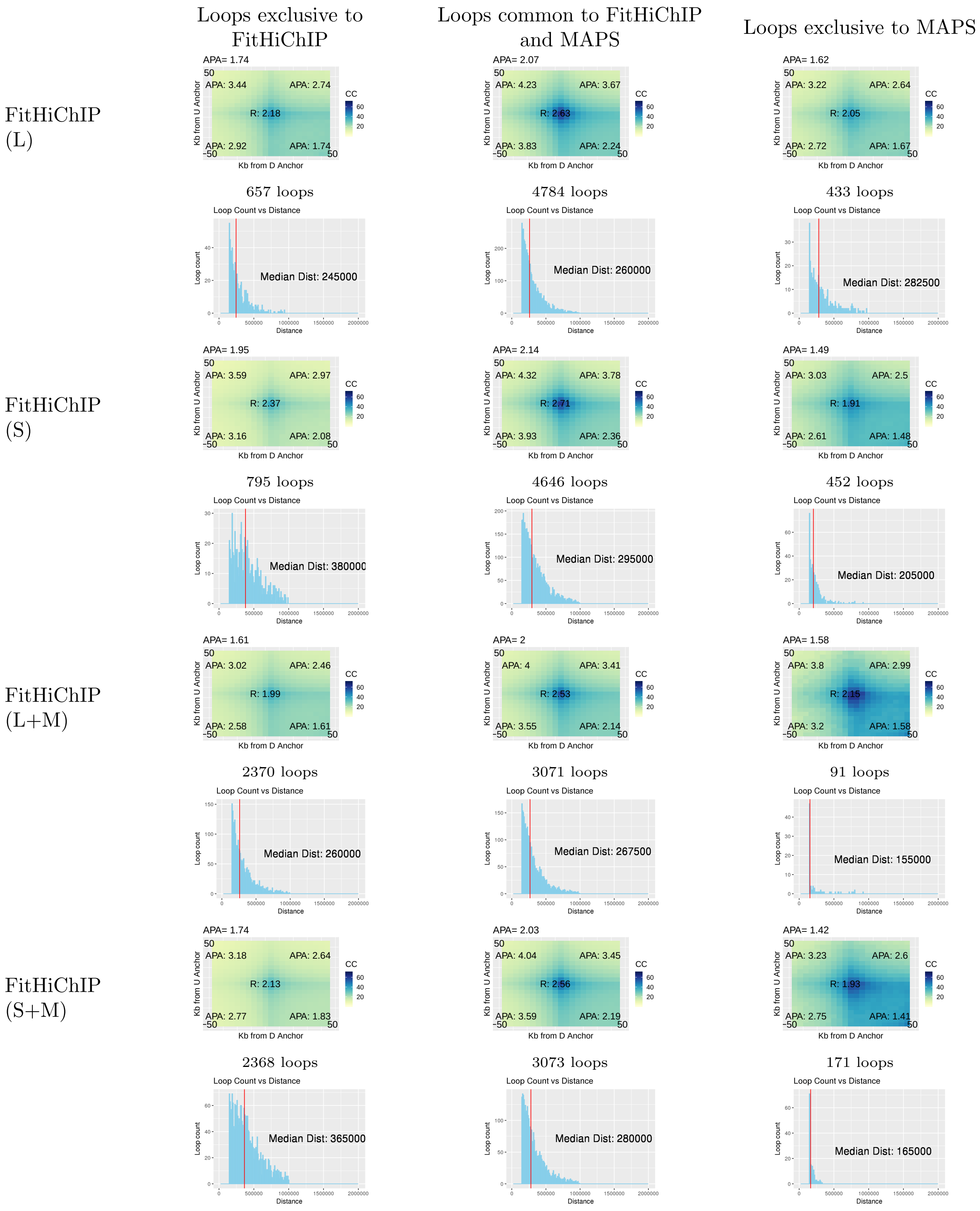

Supplementary Figure 24: **APA of overlapping and exclusive loops between FitHiChIP and MAPS for GM12878 cohesin HiChIP data [19].** Top-k loops of FitHiChIP (k=5441 within 150kb - 1Mb distance range, as mentioned in Fig. 20) show $\sim$ 80% to 90% overlap with top-k MAPS loops. However, applying merging filter reduces this overlap to $\sim$60%, since the cluster of significant loops (detected by MAPS) are replaced by a few representative loops in FitHiChIP after merged filtering. Exclusive FitHiChIP loops have higher APA scores in each case and also mostly have higher loop distances compared to exclusive MAPS loops for this dataset. When merging filter is employed in FitHiChIP, number of exclusive FitHiChIP loops is considerably higher than the number of exclusive loops in MAPS.

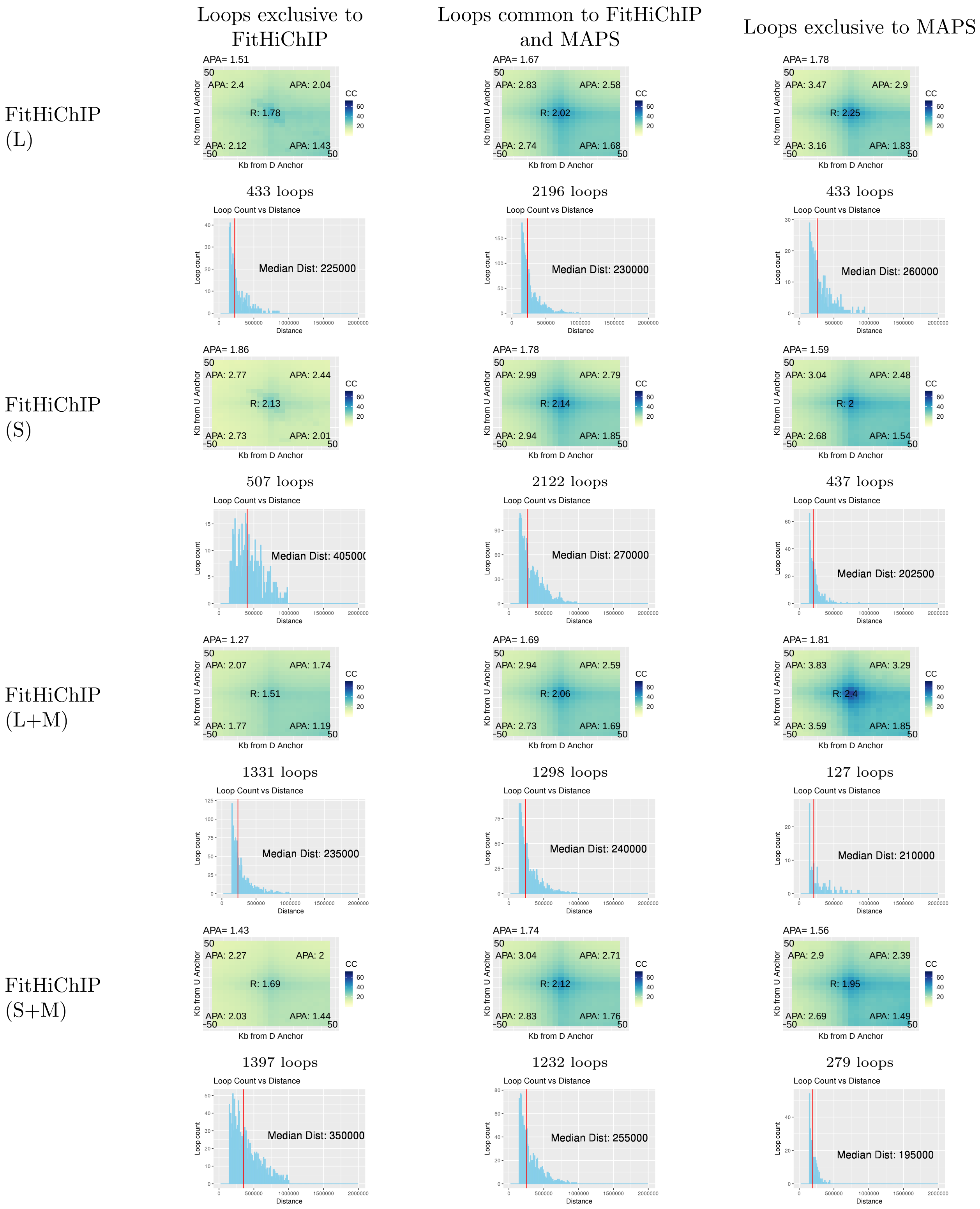

Supplementary Figure 25: **APA of overlapping and exclusive loops between FitHiChIP and MAPS for GM12878 H3K27ac HiChIP data [20].** Top-k loops of FitHiChIP (k=2629) show $\sim$ 80% overlap with top-k MAPS loops. However, applying merging filter reduces this overlap to ~50%, since the cluster of significant loops (detected by MAPS) are replaced by a few representative loops in FitHiChIP after merged filtering. Exclusive FitHiChIP (particularly with stringent background) loops have mostly lower APA scores but also higher loop distances compared to exclusive MAPS loops for this dataset. When merging filter is employed in FitHiChIP, number of exclusive FitHiChIP loops is considerably higher than the number of exclusive loops in MAPS.

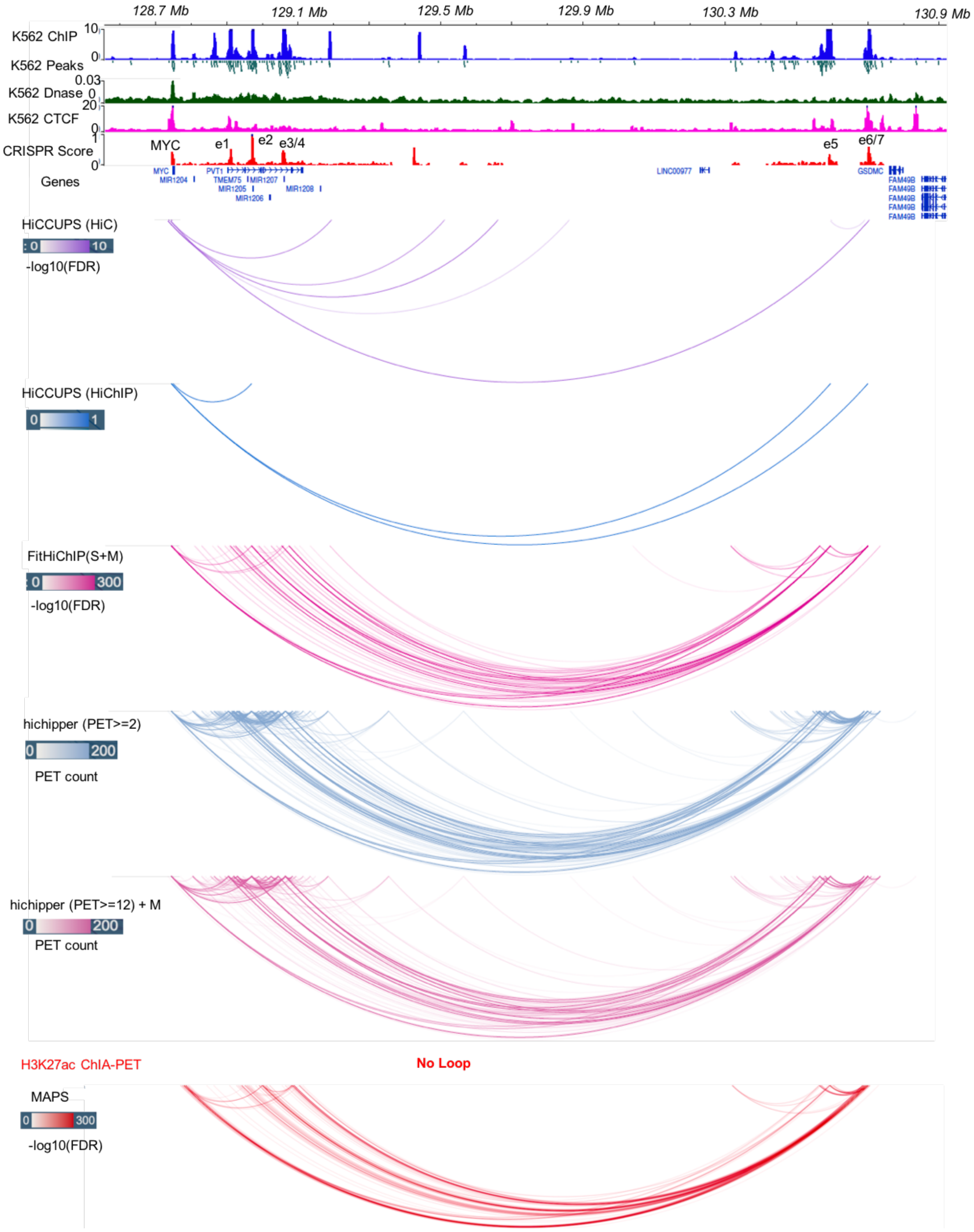

Supplementary Figure 26: **FitHiChIP recovers interactions between *MYC* promoter and seven distal CRISPRi validated enhancers [7].** Four of these enhancers were within the viewpoint presented in main text. the remaining three within ∼2Mb from the promoter region are shown here together with loop calls from K562 H3K27ac HiChIP data [20]. HiCCUPS misses loops to some of these enhancers, while hichipper results lack specificity. MAPS also detects these loops with similar specificity as FitHiChIP.

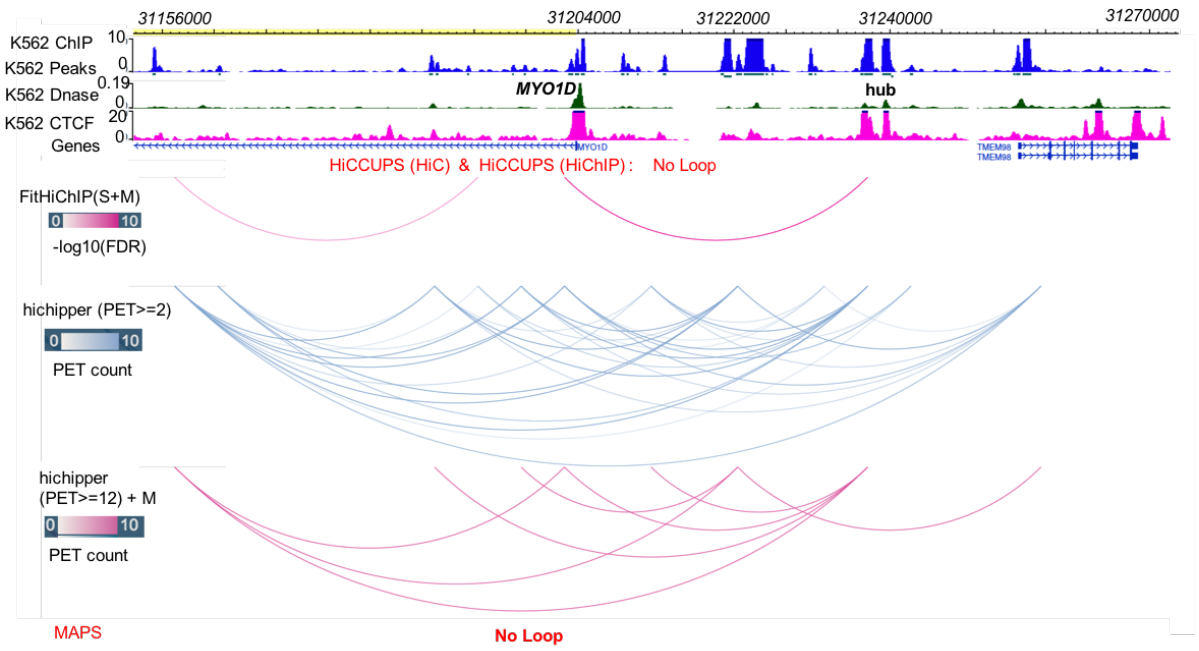

Supplementary Figure 27: **FitHiChIP recovers an interaction between the *MYO1D* promoter and an intergenic *hub* enhancer.** In all stringency settings and with or without merging, FitHiChIP identifies the loop validated in [10] between *MYO1D* and a *hub* enhancer that has the highest impact on its expression for the K562 H3K27ac HiChIP data [20]. Both HiCCUPS [20] and H3K27ac ChIA-PET [9] miss this loop. *hichipper* calls are highly non-specific. MAPS also does not detect this loop.

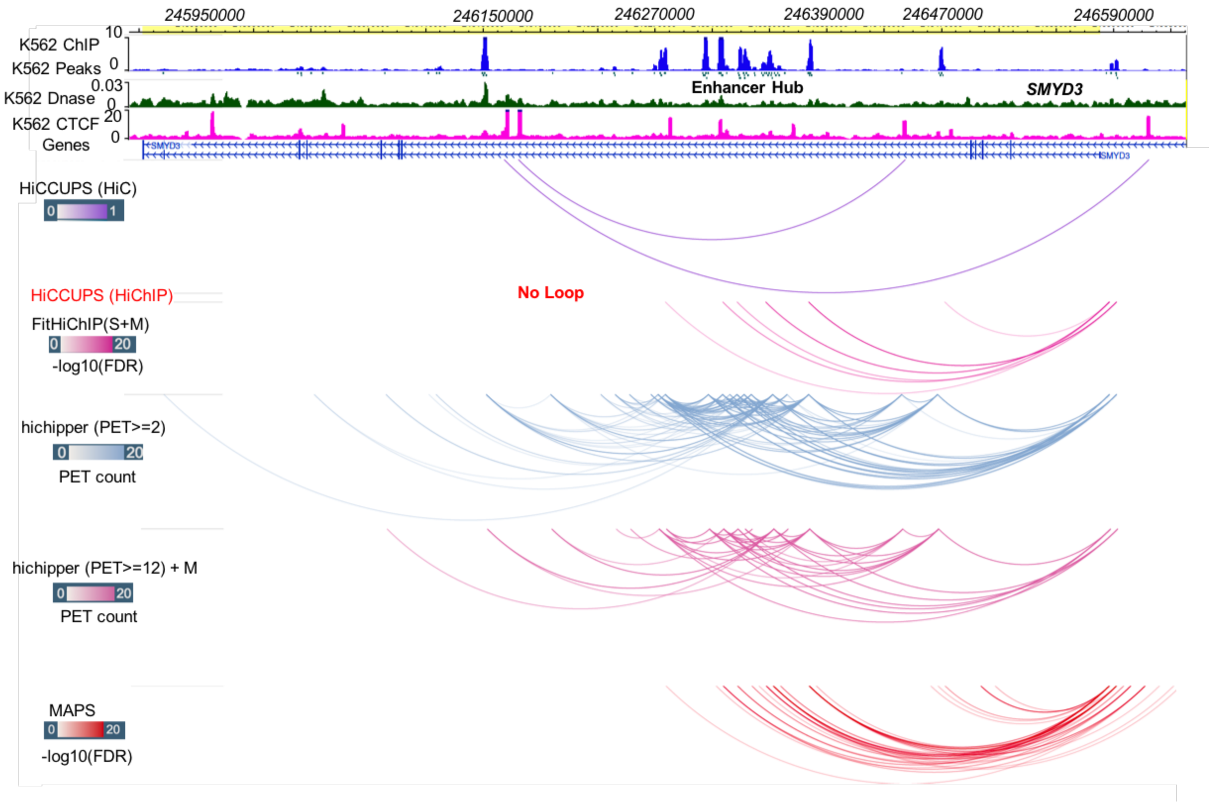

Supplementary Figure 28: **FitHiChIP recovers an interaction between the *SMYD3* promoter and an intronic *hub* enhancer.** In all stringency settings and with or without merging, FitHiChIP identifies the loop validated in [10] between *SMYD3* and a *hub* enhancer within the gene that has the highest impact on its expression for the K562 H3K27ac HiChIP data [20]. Reference HiCCUPS loops (either from Hi-C [23] or HiChIP [20] data) do not capture this interaction. The loop is missed by H3K27ac ChIA-PET [9], and RNA Pol-II ChIA-PET [4] also (not shown here). *hichipper* calls are non-specific. MAPS loops are comparable with FitHiChIP.

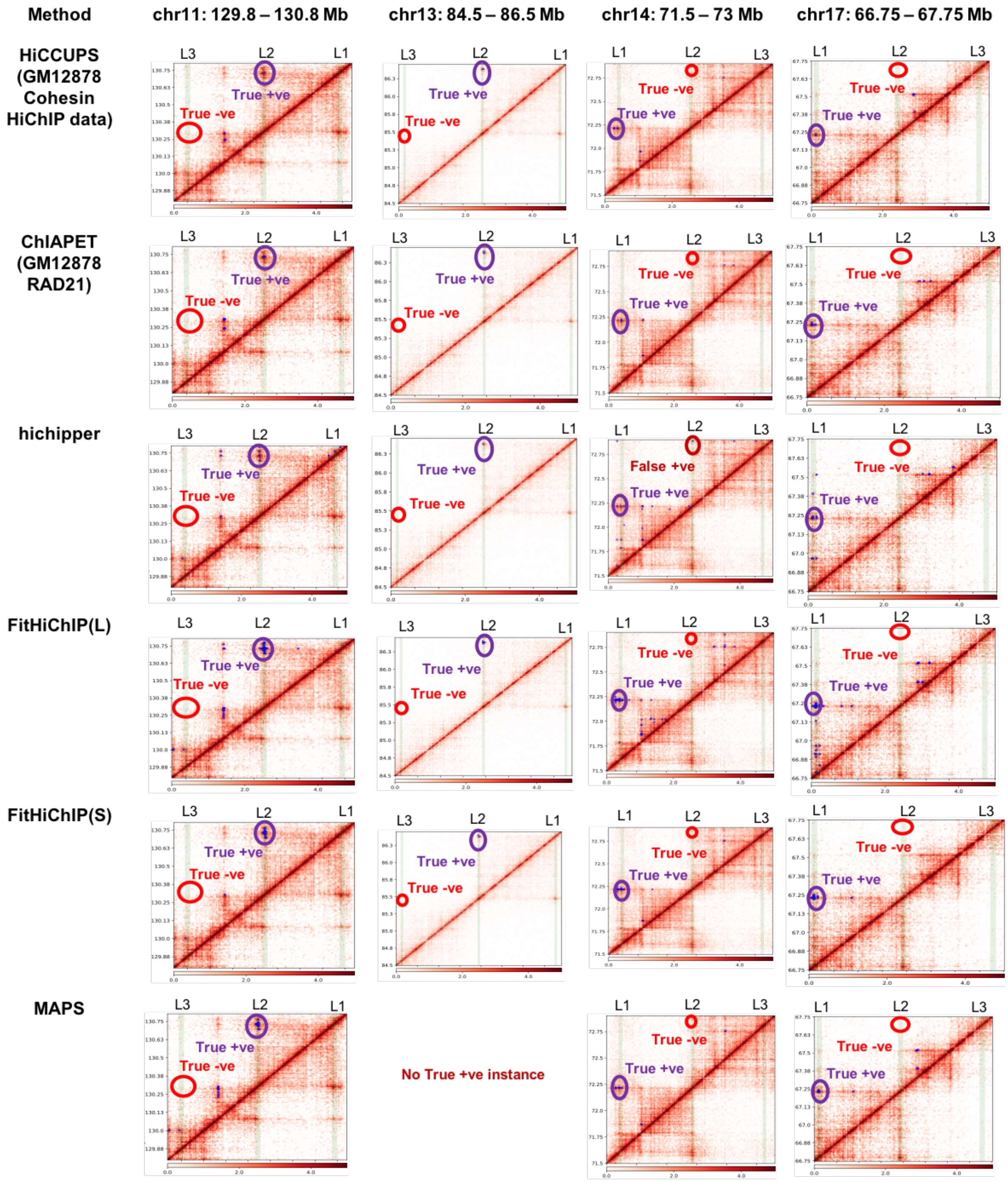

Supplementary Figure 29: **FitHiChIP captures DNA-FISH validated interactions (obtained from GM12878 in-situ Hi-C data [23]) when applied on GM12878 cohesin HiChIP data [19].** Columns 2 to 5 correspond to different chromosome regions each having three loci, namely L1, L2, and L3, where locus pairs L1 and L2 are shown to colocalize whereas the locus pairs L2 and L3 do not [23]. Methods FitHiChIP, HiCCUPS, ChIA-PET, hichipper and MAPS all recover the validated interactions (true +ve) and do not report the others (true -ve), except for chr14 where hichipper reports a false positive loop, and for chr13 where MAPS does not detect the true positive loop.

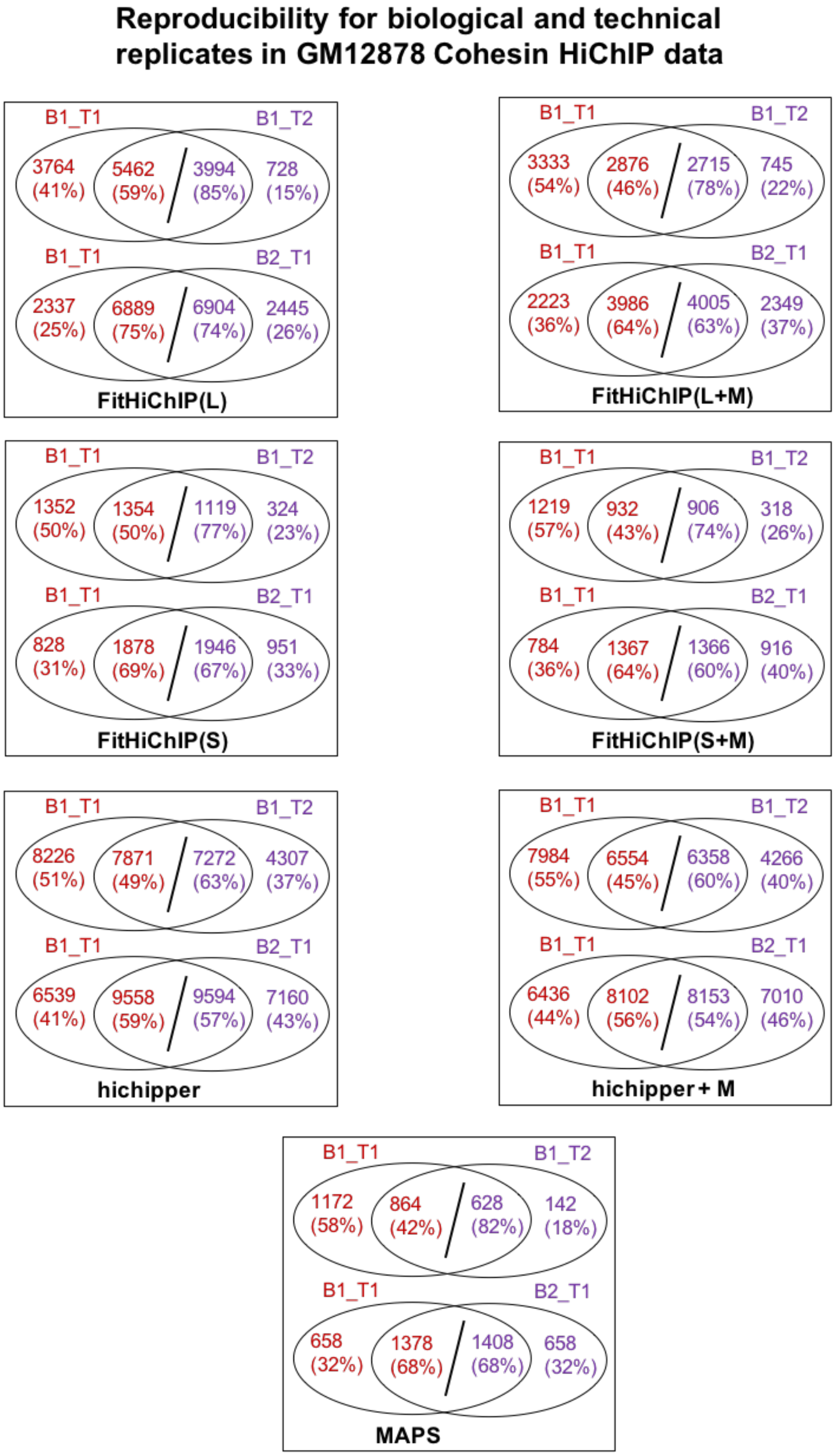

Supplementary Figure 30: **Reproducibility of loop calls across replicates for GM12878 Cohesin HiChIP dataset [19]**. Here, both FitHiChIP(L) and FitHiChIP(S) exhibit higher reproducibility than hichipper, when loop calls between technical or biological replicates are compared. Employing the merging filter retains this observation. Overlapping loops are determined using 5kb slack, and overlap with respect to individual samples are separately shown. Different colors indicate overlap of corresponding samples; counts for different samples differ due to the slack employed. MAPS shows similar reproducibility as FitHiChIP(S). Note that the total number of loop calls are likely to impact reproducibility computations, hence, FitHiChIP(L) is more comparable to hichipper results in general, and FitHiChIP(S) is more comparable to MAPS results in general.

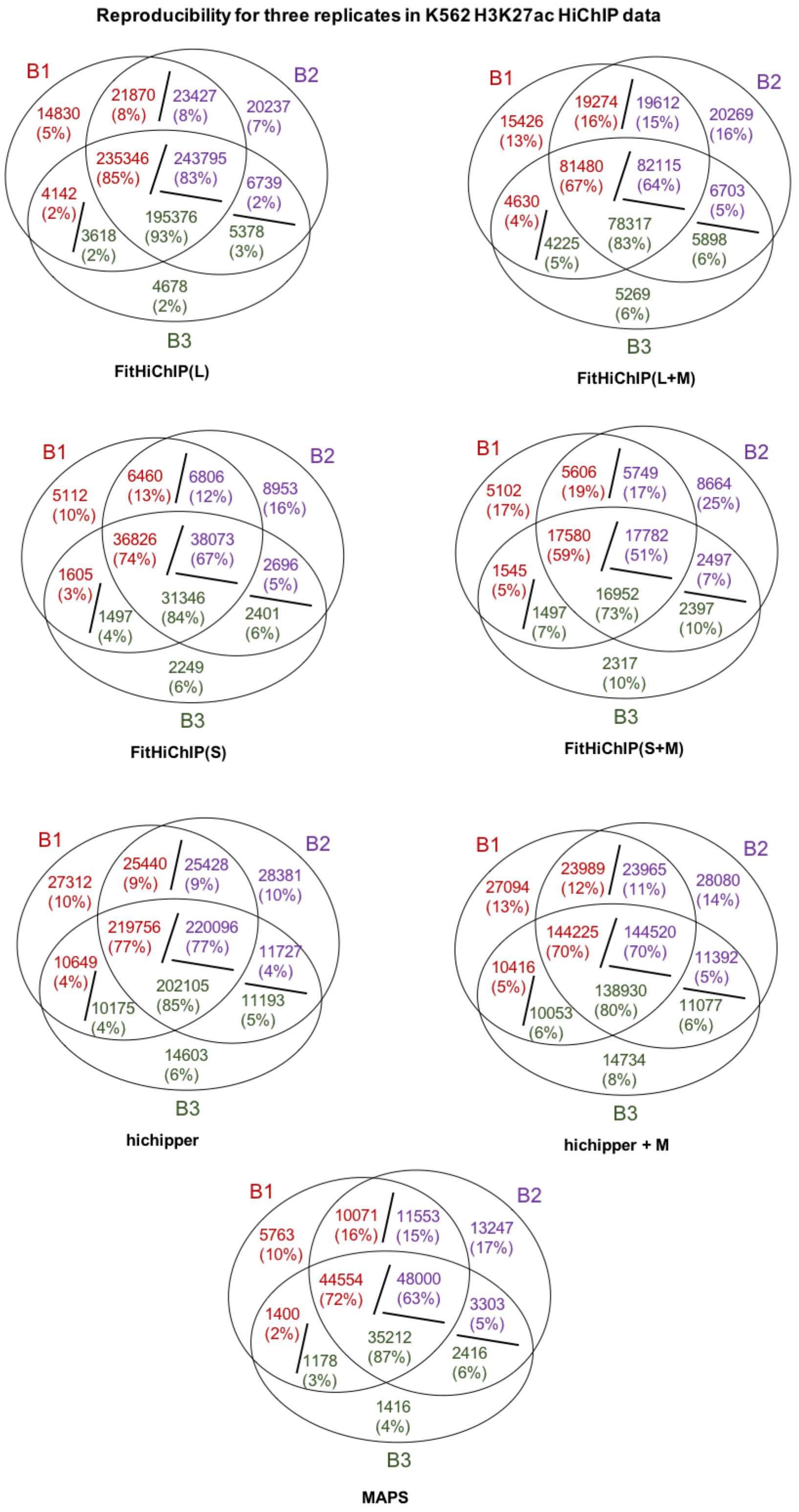

Supplementary Figure 31: **Reproducibility of loop calls across replicates for K562 H3K27ac HiChIP dataset [20]** having three biological replicates. Here, FitHiChIP(L) shows higher reproducibility than hichipper, while FitHiChIP(S) shows comparatively lower reproducibility. Employing the merging filter retains this observation. Overlapping loops are determined using 5kb slack, and overlap with respect to individual samples are separately shown. Different colors indicate overlap of corresponding samples; counts for different samples differ due to the slack employed. MAPS shows similar reproducibility as FitHiChIP(S). Note that the total number of loop calls are likely to impact reproducibility computations, hence, FitHiChIP(L) is more comparable to hichipper results in general, and FitHiChIP(S) is more comparable to MAPS results in general.

a Reproducibility of loops for mES Cohesin HiChIP data – 1M, 5M and 10M cells

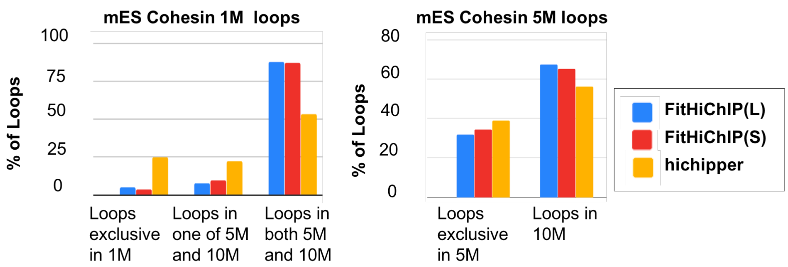

	1M	5M	10M
FitHiChIP(L)	804	7491	5954
FitHiChIP(S)	245	2351	1975
hichipper	4118	10112	9843

b Reproducibility of loops for mES H3K27ac HiChIP data – 50K, 100K, 500K and 25M cells

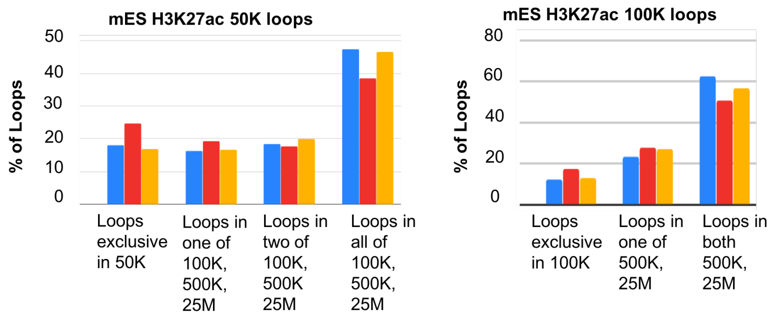

	50K	100K	500K	25M
FitHiChIP(L)	17089	13230	13850	33035
FitHiChIP(S)	2362	1636	1524	3725
hichipper	9146	8183	5970	13598

Supplementary Figure 32: **FitHiChIP shows high reproducibility of loops when HiChIP datasets from different cell counts are compared. (a)**: Analyzing reproducibility of FitHiChIP(L), FitHiChIP(S) and hichipper loops for mES Cohesin HiChIP datasets [19] of 1M, 5M and 10M cell counts (merged replicates) [19]. Reproducibility of loops between samples with lower cell counts and the samples with higher cell counts are reported. **(b)**: Similar analysis as (a) for mES H3K27ac HiChIP experiments [20] done with 50K, 100K, 500K and 25M cells.

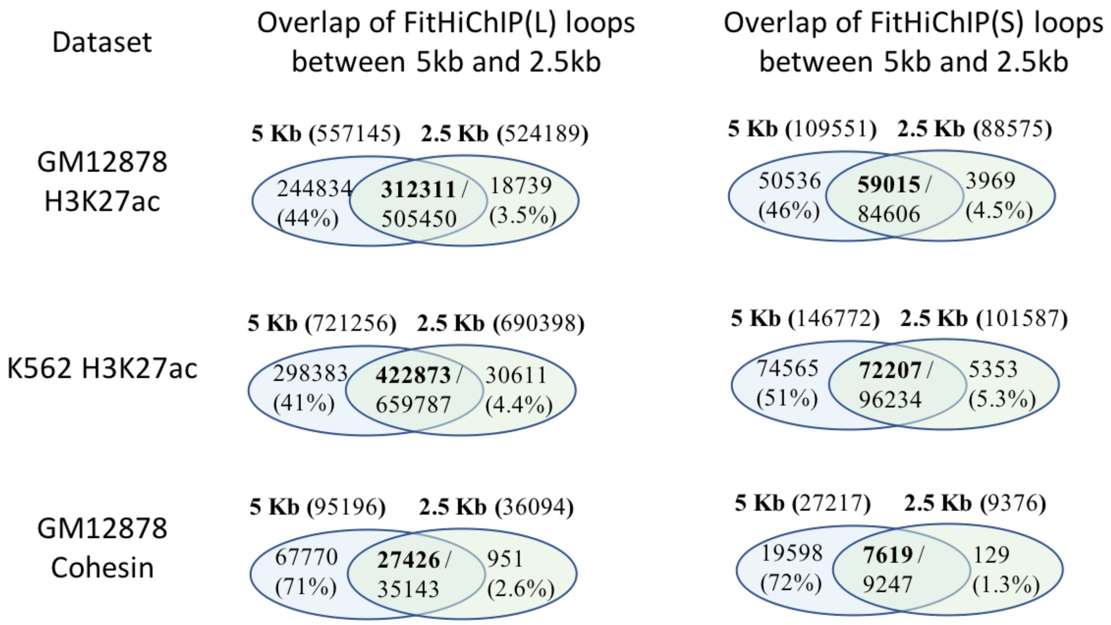

Supplementary Figure 33: **Overlap of loop calls from FitHiChIP executed with 2.5kb and with 5kb resolution contact maps for three different HiChIP datasets.** For each Venn diagram, the intersection numbers are reported with respect to the number of 5kb **(bold)** and 2.5kb (not bold) loop calls. Exact overlap (without any slack) is employed. The percentages for exclusive portions are computed using the number listed in that portion divided by all loop calls of that set listed on the top.

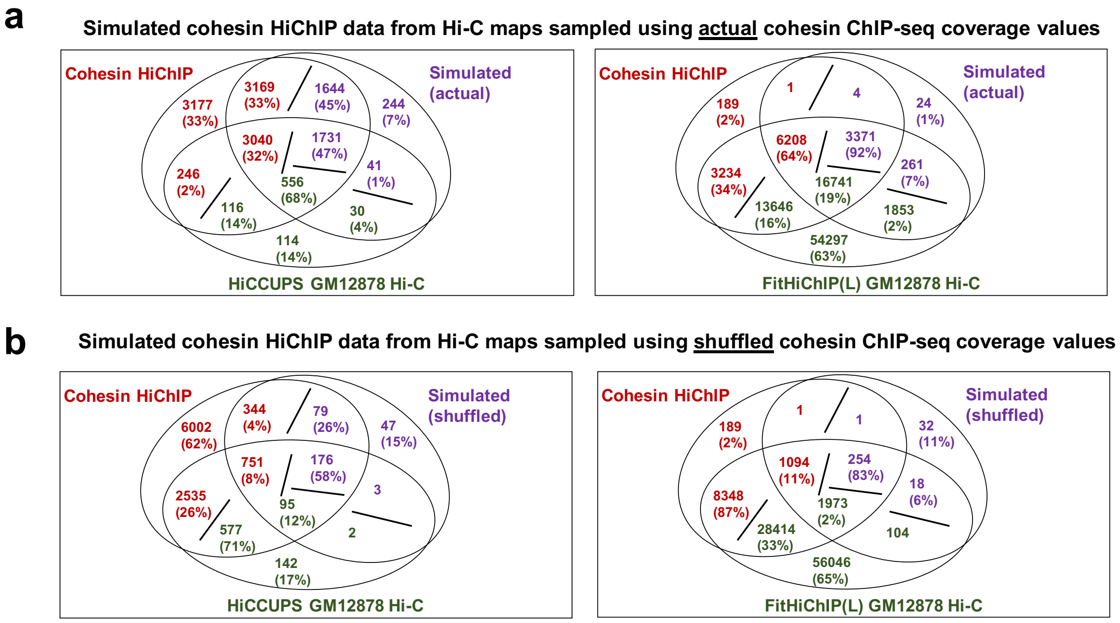

Supplementary Figure 34: **Overlap of loop calls from simulated Cohesin HiChIP maps with those from *in situ* Hi-C and real HiChIP data of GM12878.** All studied loops are within 20kb - 2Mb distance range and all results shown are for chromosome 1 only. HiCCUPS loops are filtered to have at least one interacting bin overlapping with the reference ChIP-seq peaks (peak-to-all). Loop overlap is computed using 5kb slack as described in Methods and overlap with respect to each set is reported separately for the three-way comparisons. For all applications FithiChIP(L) uses *peak-to-all* foreground (i.e., does not assign significance to pairs of non-peak bins). **(a) - left**: Three-way overlap between FitHiChIP(L) loops on GM12878 Cohesin HiChIP data [19], simulated H3K27ac HiChIP data generated and HiCCUPS Hi-C loops from GM12878. **(a) - right**: Similar overlap analysis but here FitHiChIP(L) is used for loop calling from GM12878 Hi-C data instead of HiCCUPS. **(b) - left**: Same analysis as **(a)** with the simulated HiChIP data generated by random shuffling of ChIP-seq coverage values among all 5kb bins. Peak/non-peak identity of each bin is preserved in order to study the impact of ChIP enrichment differences rather than genomic locations (e.g., overlap with promoters). **(b) - right**: Similar analysis with the simulated HiChIP data generated by random shuffling.

a Simulated H3K27ac HiChIP data from Hi-C maps sampled using actual H3K27ac ChIP-seq coverage values

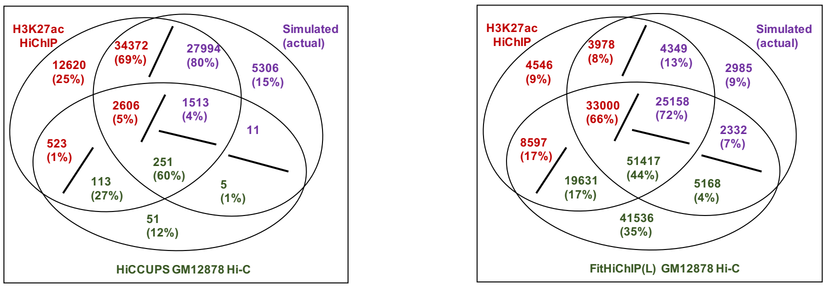

b Simulated H3K27ac HiChIP data from Hi-C maps sampled using shuffled H3K27ac ChIP-seq coverage values

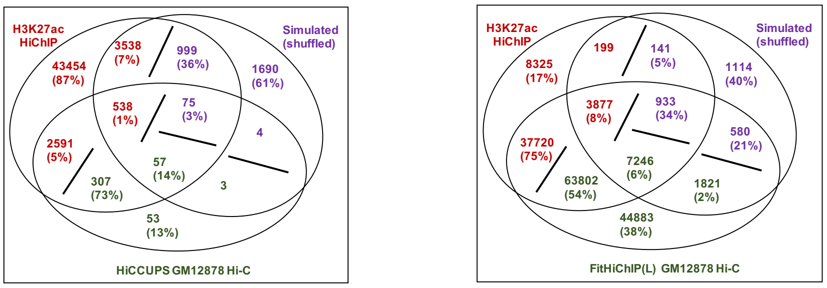

Supplementary Figure 35: **Overlap of loop calls from simulated H3K27ac HiChIP maps with those from *in situ* Hi-C and real HiChIP data of GM12878.** All studied loops are within 20kb - 2Mb distance range and all results shown are for chromosome 1 only. HiCCUPS loops are filtered to have at least one interacting bin overlapping with the reference ChIP-seq peaks (peak-to-all). Loop overlap is computed using 5kb slack as described in Methods and overlap with respect to each set is reported separately for the three-way comparisons. For all applications FithiChIP(L) uses *peak-to-all* foreground (i.e., does not assign significance to pairs of non-peak bins). **(a) - left**: Three-way overlap between FitHiChIP(L) loops on GM12878 H3K27ac HiChIP data [20], simulated H3K27ac HiChIP data generated and HiCCUPS Hi-C loops from GM12878. **(a) - right**: Similar overlap analysis but here FitHiChIP(L) is used for loop calling from GM12878 Hi-C data instead of HiCCUPS. **(b) - left**: Same analysis as **(a)** with the simulated HiChIP data generated by random shuffling of ChIP-seq coverage values among all 5kb bins. Peak/non-peak identity of each bin is preserved in order to study the impact of ChIP enrichment differences rather than genomic locations (e.g., overlap with promoters). **(b) - right**: Similar analysis with the simulated HiChIP data generated by random shuffling.

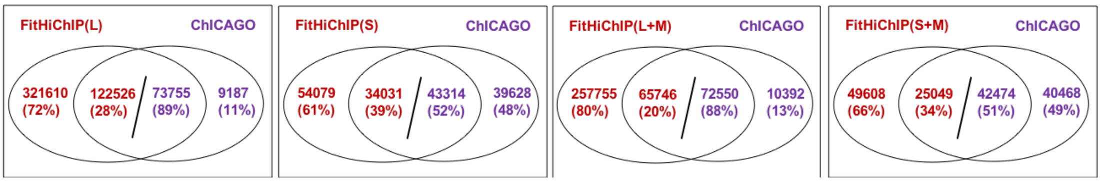

(a) Overlap of FitHiChIP (different settings) and CHiCAGO loops computed from GM12878 promoter capture Hi-C data, subject to 5 Kb slack. Number of overlapping and exclusive loops are computed with respect to the union set of loops for the corresponding datasets.

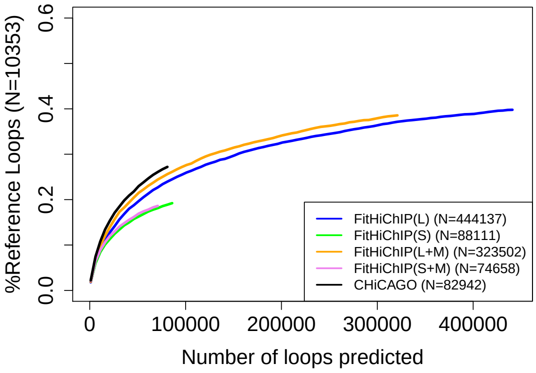

(b) **Dataset:** GM12878 PCHiC data. **Reference:** GM12878 RAD21 ChIAPET loops

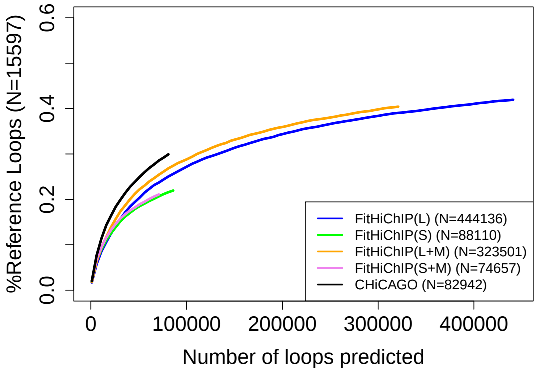

(c) **Dataset:** GM12878 PCHiC data. **Reference:** FitHiChIP(S+M) loops from GM12878 cohesin HiChIP data

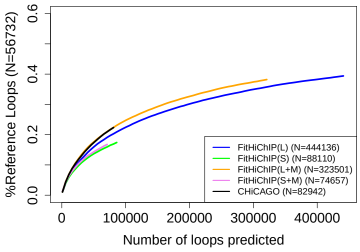

(d) **Dataset:** GM12878 PCHiC data. **Reference:** FitHiChIP(S+M) loops from GM12878 H3K27ac HiChIP data

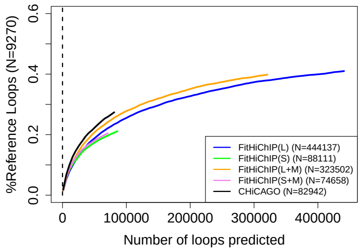

(e) **Dataset:** GM12878 PCHiC data analyzed by FitHiChIP and CHiCAGO. **Reference:** HiCCUPS loops from GM12878 Hi-C.

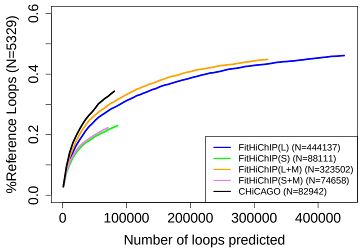

(f) **Dataset:** GM12878 PCHiC data analyzed by FitHiChIP and CHiCAGO. **Reference:** HiCCUPS loops from GM12878 H3K27ac HiChIP.

Supplementary Figure 36: **FitHiChIP applied on GM12878 promoter capture Hi-C data [18], shows high overlap with CHiCAGO loops [3]. (a)**: Venn diagrams showing the number of loops overlapping between CHiCAGO and each different setting of FitHiChIP when applied on PCHiC data. Loop overlap is computed using 5kb slack. Percentages of overlap with respect to individual samples are separately shown. **(b)-(d)**: Recovery plots of loops called from PCHiC data with different methods when we use either RAD21 ChIA-PET calls or FitHiChIP calls on the cohesin or H3K27ac HiChIP data as a reference set. Only loops within 20 Kb - 2 Mb are considered similar to HiChIP results. **(e-f)**: Comparison of CHiCAGO and FitHiChIP in recovering reference HiCCUPS loops either from Hi-C or H3K27ac HiChIP, when applied on GM12878 PCHiC data [3].

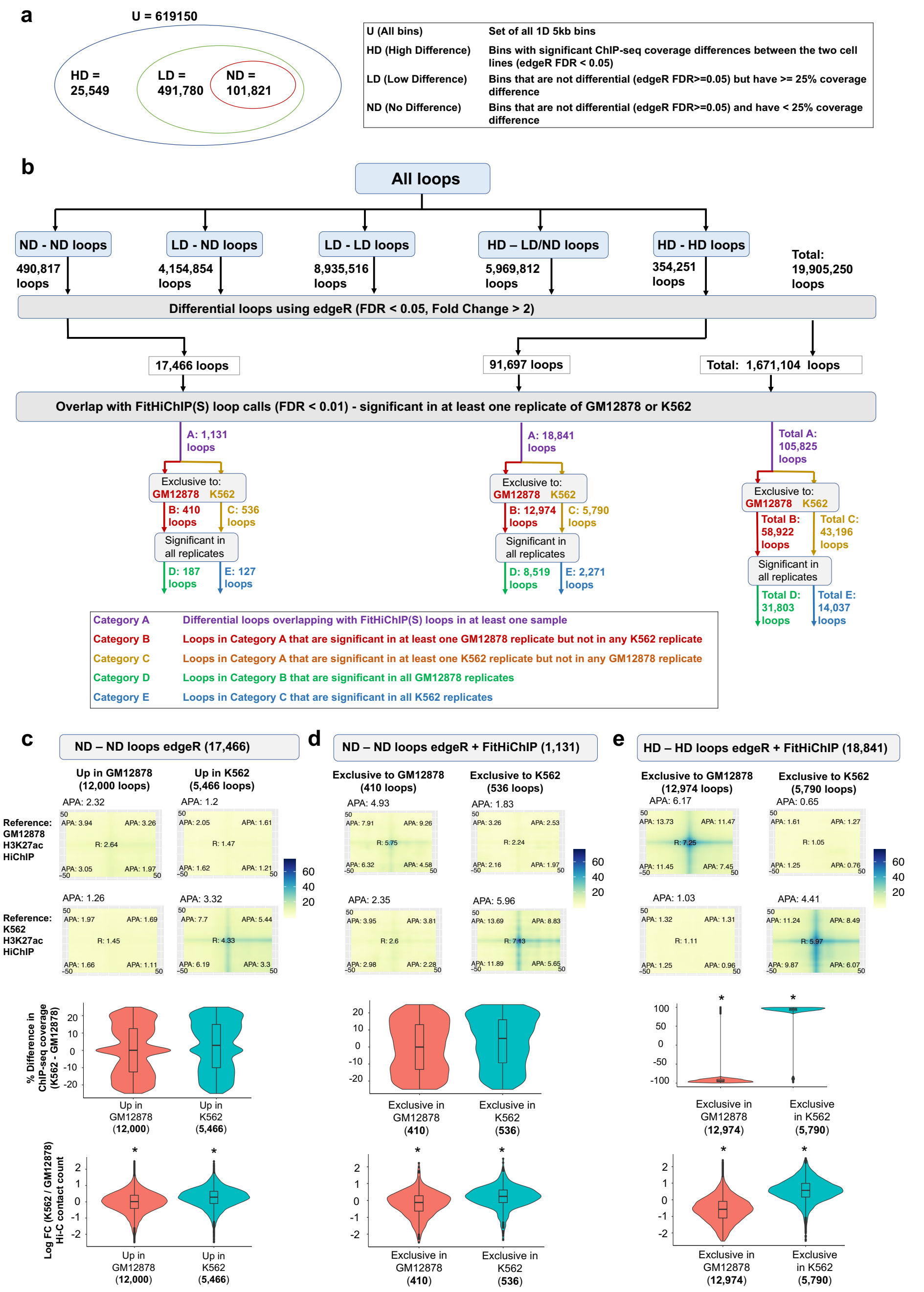

Supplementary Figure 37: **FitHiChIP supports finding differential HiChIP loops.** **(a)**: Categorization of 1D bins according to their difference in ChIP coverage for GM12878 and K562 H3K27ac ChIP-seq data (mentioned in Supplementary Table 1). **(b)**: Workflow of finding differential HiChIP loops from GM12878 H3K27ac HiChIP (two replicates) and K562 H3K27ac HiChIP (three replicates) data [20]. Differential loops (declared by EdgeR) and overlapping with FitHiChIP(S) loops significant in at least one input sample are reported. Loops exclusive to GM12878 or K562 (significant in only that category) are also shown. **(c)**: (Top) APA with respect to contact maps of merged HiChIP replicates for the differential loops (up in either GM12878 or K562 according to the log fold change of edgeR) whose interacting bins are both from the category ND. These loops may or may not overlap with FitHiChIP(S) significant loops. (Middle) The distributions of percent difference in 5kb binned ChIP-seq coverage values (K562 minus GM12878) for bins involved in loops in each category. "*" denotes a statistically significant difference for a one sample t-test for mean of the absolute difference being less than 5%. (Bottom) The distributions of log fold change of contact counts (K562 / GM12878) of these differential loops with respect to *in-situ* Hi-C data [23]. "*" denotes a statistically significant difference for a one sample t-test for mean of the log2 fold change being equal to zero. Error bars (boxplots within violin plots) represent interquartile range. **(d)**: Same as **(c)** but for the differential ND-ND loops overlapping with the FitHiChIP(S) loops significant in at least one input sample. **(e)**: Same as **(d)** but for HD-HD loops instead of ND-ND.

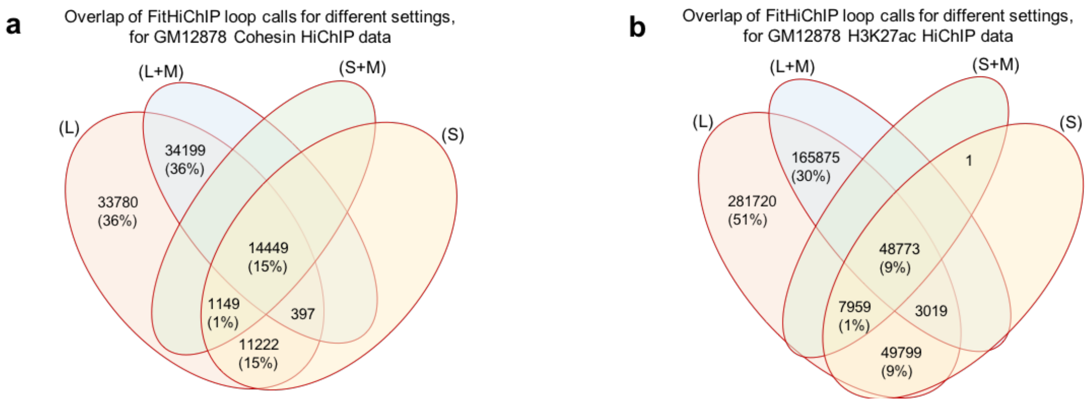

Supplementary Figure 38: **Overlap of FitHiChIP loop calls for different settings. (a)**: A 4-way overlap of loop counts for different FitHiChIP settings, using zero slack (exact overlap), for GM12878 Cohesin HiChIP data [19]. A large fraction of FithiChIP(S+M) loops are also identified by all other settings. **(b)**: Same analysis with respect to FitHiChIP loop calls from GM12878 H3K27ac HiChIP data [20].

Supplementary Tables

HiChIP Datasets	GEO Accession	Reference ChIP-seq peaks
GM12878 cohesin [19]	**GSE80820** - merged replicates: GSM2138324, GSM2138325, GSM2138326 and GSM2138327	experiments/ENCSR000DZP **Peaks**: ENCFF686FLD.bed **Align**: ENCFF797XWM.bam `www.encodeproject.org`
mES cohesin [19]	**GSE80820** - merged replicates: GSM2138328, GSM2138329, GSM2138340 and GSM2138331	experiments/ENCSR000ETL **Peaks**: ENCFF001YKF.bed `www.encodeproject.org`
mES cohesin with varying cell counts [19]	**GSE80820** - merged replicates **10M cells**: GSM2138332, GSM2138333; **5M cells**: GSM2138334, GSM2138335; **1M cells**: GSM2138336, GSM2138337	Same as above.
mES H3K27ac with varying cell counts [20]	**GSE101498** - merged replicates: **50K cells**: GSM2705039, GSM2705040; **100K cells**: GSM2705037, GSM2705038; **500K cells**: GSM2705035, GSM2705036; **25M cells**: GSM2705031, GSM2705032, GSM2705033 and GSM2705034	experiments/ENCSR000CCH **Peaks**: ENCFF001XZL.bed `www.encodeproject.org`
GM12878 H3K27ac [20]	**GSE101498** - merged replicates: GSM2705041, GSM2705042	experiments/ENCSR000AKC **Peaks**: ENCFF465WTH.bed **Align**: ENCFF197QHX.bam `www.encodeproject.org`
K562 H3K27ac [20]	**GSE101498** - merged replicates: GSM2705043, GSM2705044 and GSM2705045	experiments/ENCSR000AKP **Peaks**: ENCFF578UBP.bed `www.encodeproject.org`
CD4 naïve H3K27ac [20]	**GSE101498** - merged replicates: GSM2705048, GSM2705049, GSM2705050, GSM2705051 and GSM2705052	MACS2 generated peaks from GSM772859 ChIP-seq sample MACS2 options: -q 0.01 –extsize 147 –nomodel

Supplementary Table 1: **A list of published HiChIP datasets and reference ChIP-seq peak files from matching experiments used in this work.**

**Method**	**GM12878 H3K27ac HiChIP**		**K562 H3K27ac HiChIP**		**GM12878 cohesin HiChIP**		**mES cohesin HiChIP**	
	Median distance (kb)	Number of loops	Median distance (kb)	Number of loops	Median distance (kb)	Number of loops	Median distance (kb)	Number of loops
HiCCUPS	120	5329	110	1383	165	9564	165	7696
ChIA-PET	-	-	70	1738	165	10353	125	17540
hichipper (PET ≥ 2)	235	305911	295	484804	160	52590	130	27765
hichipper (PET ≥ 12)	100	70519	135	132757	100	3654	75	1306
hichipper (PET ≥ 2) + M	280	234279	355	337110	170	45667	140	22957
MAPS	165	185167	175	245491	160	38894	-	-
FitHiChIP(L)	150	557145	180	721256	170	95196	165	115617
FitHiChIP(L+M)	175	217666	210	285042	175	49044	170	60391
FitHiChIP(S)	200	109551	240	146772	255	27217	250	35041
FitHiChIP(S+M)	210	56732	245	80970	250	15597	250	19910

Supplementary Table 2: **Total number of loop calls and the median distance (in kb) of such loops for each method and each HiChIP dataset.** HiCCUPS loop calls at a mixture of 5kb and 10kb resolution are gathered as described in Supplementary Table 3. ChIA-PET loops listed in Supplementary Table 4 and hichipper loops called by several different settings as described in Methods are all mapped to 5kb bins (see Methods). FitHiChIP is run on data with all replicates merged as described in Supplementary Table 1 and binned at 5kb resolution using coverage bias correction and peak-to-all pairs as the foreground set. MAPS loops are computed using all replicates. All of the HiChIP loop calling methods FitHiChIP, hichipper and MAPS use as input the ChIP-seq peak calls from matching datasets as listed in Supplementary Table 4. The reported numbers are from loop calls within a distance range of 20kb - 2Mb.

Dataset	Source
HiCCUPS loops computed from the HiChIP datasets listed in Supplementary Table 1)	Gathered from Mumbach et al. 2016 and 2017 [19, 20].
HiCCUPS loops computed from GM12878 (primary+replicate) and K562 *in-situ* Hi-C data	Gathered from Rao et al. 2014 [23].

Supplementary Table 3: **HiCCUPS loops used in the current study. Only the loops within a distance range of 20kb - 2Mb are considered.**

Dataset	Source
GM12878 RAD21 ChIA-PET (used to benchmark GM12878 cohesin HiChIP data)	Gathered from Heidari et al. 2014 [9]
mES Smc1a ChIA-PET (used to benchmark mES cohesin HiChIP data)	Gathered from Dowen et al. 2014 [5]
K562 H3K27ac ChIA-PET (used to benchmark K562 H3K27ac HiChIP data)	Gathered from Heidari et al. 2014 [9]
K562 Pol2 ChIA-PET (used to benchmark K562 H3K27ac HiChIP data)	Gathered from Cao et al. 2017 [4], generated by Li et al. 2012 [15]

Supplementary Table 4: **ChIA-PET loops used in the current study. All loops are mapped with respect to nearest 5kb bin. Only the loops within a distance range of 20kb - 2Mb are considered.**

Dataset	Source
CD4 naïve Promoter Capture Hi-C interactions with ChiCAGO [3] score ≥ 5	Gathered from Javierre et al. 2016 [12]
GM12878 Promoter Capture Hi-C datasets (fastq files, merged replicates) from GEO:GSE81503. Data is processed as described in Methods. Interactions with ChiCAGO score ≥ 5 on the merged data are used as reference loop calls.	Gathered from Mifsud et al. 2015 [18]

Supplementary Table 5: **Promoter capture Hi-C loops and datasets used in the current study. Only the loops within a distance range of 20kb - 2Mb are considered.**

Supplementary Notes

Supplementary Note 1: Regression on bias values improves FitHiChIP loop calling

In order to normalize for coverage differences across different genomic bins due to known bias factors such as number and length of restriction fragments, GC content and mappability, FitHiChIP employs a regression model that learns the dependency of observed contact counts for each equal occupancy bin and bias values computed either by matrix-balancing-based methods (e.g., ICE [11]) or simply by coverage values normalized separately for bins that overlap peaks and for bins that do not **(See Methods)**. Compared to not using normalization, using either set of bias values for regression performs better in terms of recovering reference sets of Hi-C, ChIA-PET and PCHiC loops, highlighting the importance of accounting for such coverage differences **(Fig. 1(b), Supplementary Figure 4)**. When compared to one another, the two different bias corrections perform similarly in recovering reference loops and in terms of enrichment scores computed by APA plots **(Supplementary Figures 4 - 5)**. However, since ICE assumes all regions should have equal visibility regardless of their peak status, it introduces artifacts for several low coverage non-peak regions of HiChIP data **(Supplementary Figure 6)**. In order to avoid such artifacts and because computation of coverage bias is much simpler compared to ICE normalization, we use coverage bias correction throughout this work. Accordingly, the default and suggested setting of FitHiChIP is to use coverage bias values for achieving normalization.

Supplementary Note 2: Improvement in loop calls using a merging filter

FitHiChIP also supports merge based filtering of adjacent significant loops by applying a connected component model (see **Methods, Supplementary Figure 1** for a detailed description of *merging filter* algorithm) to eliminate the indirect contacts from a pair of highly interacting regions with dense distribution of significant loops to improve our loop detection. Note that this filtering (denoted by **+M**) is applicable to loop calls from FitHiChIP as well as other existing methods. When we employ this merging filter, we observe a significant improvement in specificity for FitHiChIP (both L and S) in terms of recovering loops from *in situ* Hi-C, PCHiC, ChIA-PET and the stringent set of HiCCUPS loops from HiChIP data **(Fig. 1(c), Supplementary Figures 7- 8)**. This improvement in specificity with merging filter is also independent of whether ChIP-seq peaks or peaks inferred from HiChIP data (discussed below) are used for FitHiChIP **(Supplementary Figure 9)**. The default setting of FitHiChIP is to use the merging filter, which is strongly recommended for deeply sequenced HiChIP libraries (>300M reads) especially when the loose (peak-to-all) background is used.

Supplementary Note 3: Robustness of 1D peak calling from HiChIP data

FitHiChIP supports either using an external set of peaks inferred from ChIP-seq experiments or inferring peaks from the reads generated by the HiChIP experiment in question by running MACS2 [26]. Similarly, hichipper also runs MACS2 for peak calling but, in addition, it introduces a correction for RE site bias in HiChIP read distribution. Here we first assess the contribution of different read groups from HiChIP data to peak calling by MACS2 [26]. We use different combinations of 1) dangling end (DE), 2) self-cycle (SC), 3) re-ligation (RE), and 4) CIS short-range ($<$ 1kb) valid (V) reads [2] for peak calling **(see Methods)**. As recommended by an earlier work [19], hichipper uses DE reads plus SC reads as the input to MACS2. We test several other combinations of HiChIP reads and compute the recovery of reference ChIP-seq peaks from matching experiments to ask which combination performs better in recovery. We find that other than using only V reads (as suggested in [6]), most other combinations perform similarly including DE+SC used by hichipper and DE+SC+RE+V used by FitHiChIP as default **(Fig. 1(d), Supplementary Figure 10)**. We also show that the RE site bias correction introduced by hichipper reduces the number of overall peaks called, but does not lead to better recovery of ChIP-seq peaks in general (complete overlap with most other settings), except from GM12878 cohesin data **(Supplementary Figure 10)**. The differences between different read combinations in ChIP-seq peak recovery become even more negligible when peaks are binned at 5kb fixed-size bins to assign a peak vs non-peak status to such bins **(Supplementary Figure 11)**. The default setting of FitHiChIP when no external ChIP-seq peaks are provided is to use DE+SC+RE+V reads to find 1D peaks using MACS2 **(Methods)**.

Supplementary Note 4: Assigning peak-bins using either ChIP-seq or HiChIP peaks

Since FitHiChIP distinguishes between bins that overlap with 1D peaks of ChIP signal, we test whether peaks inferred from ChIP-seq or from HiChIP data better improve performance of FitHiChIP in recovering loop calls from other conformation capture assays. As discussed above, ChIP-seq reports a smaller number of peaks most of which are recovered by peak calls from HiChIP data. Accordingly, loop calls using ChIP-seq peaks tend to have better specificity in most cases compared to HiChIP peaks **(Fig. 1(e), Supplementary Figure 12)**.

Therefore, we utilize ChIP-seq peaks in this work since this data is readily available **(Supplementary Table 1)**. When matching ChIP-seq data is not available, and the users infer peaks from HiChIP data (a larger number compared to ChIP-seq peaks), we suggest using the stringent (S) background to compensate for the leniency resulting from the use of a large number of peaks for background estimation.

Supplementary Note 5: FitHiChIP loops show enrichment in Hi-C contact maps

Previous section shows the recovery of loops called from Hi-C and other datasets by FitHiChIP and existing methods. Here we start from HiChIP loops and ask whether they are supported by Hi-C contact maps. First, we ask what fraction of loops that are common between FitHiChIP and hichipper, or are specific to one method are identified as loops from Hi-C data either by a local (HiCCUPS [23]) or a global (FitHiC [1]) model of background estimation for detecting significant loops from Hi-C. HiCCUPS reports a small number of loops with high specificity, whereas FitHiC calls have better sensitivity but lower specificity. Hence, comparison against these two reference sets should capture overlap of HiChIP calls with the full spectrum of interactions from Hi-C data. **Supplementary Figure 18** shows that a large fraction of HiChIP loops overlap with FitHiC calls suggesting enrichment of contact counts for these pairs compared to other locus pairs with the same genomic distance. This overlap is highest for cohesin HiChIP data and for loops either common to FitHiChIP and hichipper (yellow and green), or exclusively called by FitHiChIP (blue). For both cohesin and H3K27ac, the overlap is the lowest for hichipper-specific loop calls (purple). Similar analysis for Hi-C loops identified by HiCCUPS shows much lower overlaps for all sets reflecting the stringency of HiCCUPS **(Supplementary Figure 18)**. However, the relative trend of overlap being higher for loops called by FitHiChIP (either common with hichipper or exclusive to FitHiChIP) compared to hichipper remains the same. In agreement with the recovery analysis and APA plots described below, these results suggest that hichipper loops consistently have lower support from Hi-C data compared to FitHiChIP. Similar comparison between FitHiChIP and MAPS shows that loop calls that are specific to MAPS (in comparison to FitHiChIP) are also well supported by the underlying Hi-C data unlike hichipper **(Supplementary Figure 19)**. These results suggest that both FitHiChIP and MAPS calls from HiChIP data largely correspond to regions of contact enrichment in Hi-C maps which are likely to be further amplified by the HiChIP assay.

Next, to further assess the HiChIP loop calls from different methods in terms of support from Hi-C data while controlling for their differences in stringency, we employ aggregated peak analysis (APA) [22] which aggregates local contact patterns from Hi-C data around a set of loop calls. For generating APA plots and scores, we use normalized Hi-C contact counts in 50kb up and downstream of predicted loops that are within the genomic distance range of 150kb - 1Mb (as suggested in [22]). Other than FitHiChIP, hichipper and MAPS, we conduct this analysis for HiCCUPS loops from Hi-C and HiChIP data as well as loops from matching ChIA-PET experiments as our reference points. When we perform APA for 6219 loops (150kb - 1Mb) called by HiCCUPS on GM12878 Hi-C data, we get an averaged APA score (top of the figure) of 2.17 **(Fig. 3(a))**. This high enrichment is reflective of the fact that these loop calls are from the same Hi-C data the APA plots are made. When we perform a similar analysis for 5108 HiCCUPS loops from cohesin HiChIP data [19] and for 5441 loops from RAD21 ChIA-PET data [9], we get APA scores of 1.93 and 1.83, respectively **(Fig. 3(b), Supplementary Figure 20)**. As the number of reference HiChIP HiCCUPS and ChIA-PET loops are considerably lower, we sample the top-k (k=5441) loops for all HiChIP loop callers to compute the APA scores for GM12878 cohesin HiChIP data. For this set of loops, all versions of FitHiChIP exhibit higher enrichment of center pixels (denoted as R) as well as higher averaged APA scores compared to hichipper in different settings and compared to ChIA-PET **(Fig. 3(c), Supplementary Figure 20)**. In agreement with the previously mentioned overlap analysis between MAPS and reference Hi-C loops **(Supplementary Figure 19)**, the top-k MAPS loop calls are also highly supported by Hi-C data with an APA score of 2.02 **(Fig. 3(d))**. Notably, cohesin HiChIP loops are stronger in enrichment compared to H3K27ac loops regardless of the loop prediction method **(Figs. 3(b-h) vs Supplementary Figure 21)** suggesting that cohesin bound regions are involved in well-demarcated loops likely marking the TAD/subTAD boundaries compared to H3K27ac loops that are mainly between specific regulatory elements generally within TADs. For both cohesin and H3K27ac datasets, the stringent setting of FitHiChIP reports the highest APA scores even though it has the largest median loop distance compared to other HiChIP loop calling methods, suggesting that higher APA scores are not due to preference of shorter range loops **(Supplementary Figures 20 and 21)**.

We then further evaluate the top-k loop calls (k = 5441 for GM12878 cohesin; 2629 for GM12878 H3K27ac) of FitHiChIP, hichipper and MAPS to see whether the loops exclusively predicted by one method show higher enrichment with respect to Hi-C maps (i.e., higher APA score) **(Fig. 3(i))**. For FitHiChIP and MAPS, the top-k loops are selected by higher statistical significance (lower q-values) whereas hichipper loops are filtered with respect to PET counts as significance is not reported. Considering GM12878 cohesin HiChIP dataset, loops exclusive to FitHiChIP (for all settings) show higher APA scores than the loops exclusive to hichipper, in spite of having higher median loop distances particularly for the stringent background **(Figs. 3(j-l), Supplementary Figure 22)**. The same trend follows for GM12878 H3K27ac HiChIP data **(Supplementary Figure 23)**,

suggesting that hichipper misses loops highly supported by *in-situ* Hi-C data. Comparing FitHiChIP without merging filter (FitHiChIP(L) and FitHiChIP(S)) and MAPS loops for these datasets show that majority (> 80%) of these top-k loops are common to both methods **(Supplementary Figures 24 and 25)**. When merging filter is employed (FitHiChIP(L+M) and FitHiChIP(S+M)), number of exclusive loops in FitHiChIP is much higher than those exclusively in MAPS. This is because, MAPS prioritizes loops in clusters (interacting bins are adjacent) resulting in multiple reports from a local region all with high Hi-C contact enrichment, while merging filter in FitHiChIP eliminates many among such clustered loops. This results in an asymmetry in the exclusive loop counts due to the slack given in computing overlap between any given sets of loops. Compared to FitHiChIP with merging filter, loops exclusive to MAPS have lower APA scores for GM12878 cohesin HiChIP data **(Supplementary Figure 24)** but have higher scores when H3K27ac HiChIP data is considered **(Supplementary Figure 25)**. In both cases, the number of method-specific loops are at least 10 times higher for FitHiChIP.

Supplementary Note 6: FitHiChIP identifies independently validated distal loops

To assess whether FitHiChIP connects distal enhancers to their experimentally validated target promoters from HiChIP data, we compile a list of loci for which functional data (e.g., CRISPRi) is available together with the HiChIP data for the same cell line [7, 4, 10, 8]. One gene with relatively comprehensive enhancer screening data is *MYC*, for which several megabases of surrounding region is tiled with sgRNAs for CRISPRi screen of cellular proliferation in K562 cells [7]. This screen identified seven distal regions (e1–e7) ranging from 160kb to ∼2Mb in distance to *MYC* promoter [7]. All of these regions are identified as interacting with *MYC* promoter using RNA Pol II ChIA-PET [4], hence are likely to inhibit proliferation through long-range regulation of *MYC* expression. For the ∼400kb region with e1–e4, both FitHiChIP and MAPS identify all four enhancer regions as interacting with *MYC* promoter from H3K27ac HiChIP data from K562 cells, whereas HiCCUPS (applied to H3K27ac HiChIP) [20] only reports the loop with e2 and hichipper reports a large number loops in this locus, most of which are short range and not from the *MYC* promoter **(Fig. 4(a))**. Note that it is difficult to rule out the existence of other loops that are structural or are not impacting *MYC* expression, hence, we avoid using the term false positive for such hichipper loops even though they are not supported by CRISPRi results, Hi-C loops, ChIA-PET loops or by HiChIP loops from any of the other three methods. When sorted by significance, five out of top six FitHiChIP loops either connect the *MYC* promoter to e1, e2 or e3-e4 (within the same bin) or connect enhancers to each other (e1/e2 to e3-e4). For several other H3K27ac peak regions between *MYC* and the nearest validated enhancer e1, which are much closer in genomic distance to *MYC*, FitHiChIP does not report any loop calls highlighting its accuracy in recapitulating functional links. The loops to the farthest enhancers (e5 or e6-e7 at ∼2Mb) are captured by all loop callers as well as by Hi-C **(Supplementary Figure 26)**.

Another important gene *NMU* encodes for a neuropeptide that is highly conserved in mammalian brain and is expressed in other tissues. A recent single-cell CRISPR screen in K562 cells identified a significant decrease in *NMU* expression when an enhancer that is ∼100kb upstream is inhibited by CRISPRi, similar to *MYC* example but using single-cell measurements rather than bulk [8]. When applied to K562 H3K27ac HiChIP data, FitHiChIP accurately captures this loop, whereas both HiCCUPS and MAPS fail to do so **(Fig. 4(b))**. The loop calls from hichipper involve other loops in this locus with default settings ($\geq$ 2 PETs) but are identical to FithiChIP(S+M) when a more stringent count filter ($\geq$ 12 PETs) is used together with our merging filter (M). HiCCUPS calls on K562 Hi-C data recover a similar loop reported at a lower resolution (10kb bins).

Another example is *TP53*, for which super-enhancer and broad domain analysis coupled with RNA Pol II ChIA-PET data in K562 cells identified two hit regions as potential enhancers: one ∼100kb downstream and one ∼150kb upstream of the *TP53* promoter [4]. Using EpiSwitch baits, both regions are shown to interact with the *TP53* promoter, in addition to K562, in all CML patients (9/9) and nearly all normal subjects. FitHiChIP identifies loops from *TP53* promoter to both hit regions with a small number of other loops in the domain, whereas HiCCUPS misses both **(Fig. 4(c))**. On the other hand, hichipper, both with default settings and with additional filters, as well as MAPS report a large number of other loops in this locus many including the *TP53* promoter. HiCCUPS loops from Hi-C data fail to capture either of these loops.

Another two genes for which several CRISPRi and CRISPR KO experiments are carried out in K562 cells are *MYO1D* and *SMYD3* [10]. For both genes, FitHiChIP reports the loop between the promoter and a *hub* enhancer with the highest impact on gene expression. Similar to the previous examples, HiCCUPS applied on HiChIP data fails to capture these loops for both genes and hichipper reports a large number of other loops involving these two genes even after additional filtering **(Supplementary Figures 27, 28)**. Similar to HiCCUPS, for the *MYO1D* locus, MAPS fails to detect the loop involving that promoter **(Supplementary Figure 27)** but successfully reports the loops between the *SMYD3* promoter and the large stretch of enhancers (∼200-300kb away from the promoter) within the defined enhancer hub **(Supplementary Figure 28)**. On the other hand, for *SMYD3* locus, HiCCUPS loops from Hi-C data reports a ∼450kb-long loop between two CTCF sites that spans the ∼200-300kb promoter-enhancer loops described above. This CTCF loop is not captured by

any of the HiChIP loop callers and Hi-C does not capture the promoter-enhancer loops reported from H3K27ac HiChIP data as discussed above, providing an example of different types of loops captured by Hi-C data in comparison to H3K27ac HiChIP data and vice versa.

Supplementary Note 7: FitHiChIP recovers long range interactions validated by FISH

We also test FitHiChIP, HiCCUPS, MAPS and hichipper on GM12878 cohesin HiChIP data, corresponding to four sets of loci identified from Hi-C and validated by FISH to have strong CTCF-dependent long-range loops [23]. Each set contains three loci, namely L1, L2, and L3, where L2 is equidistant from L1 and L3, but loops occur between L1 and L2, and not between L3 and L2 (negative control). All of these four methods capture almost all of the validated loops (DNA FISH between L1-L2) from GM12878 cohesin HiChIP data (aside from one false negative for MAPS on chr13) and almost none of the distance matched negative controls (aside from one false positive for hichipper on chr14), which are confirmed to not colocalize by DNA FISH **(Supplementary Figure 29)**.

Supplementary Note 8: Reproducibility and robustness of FitHiChIP loops

For several HiChIP datasets with technical and biological replicates, we ask the question to what extent loops identified from different replicates overlap with each other when different loop calling methods are used **(Fig. 5(a), Supplementary Figures 30, 31)**. Considering GM12878 H3K27ac HiChIP data which has two biological replicates, 92% to 93% of FitHiChIP(L) loops are common, whereas this numbers are between 85% to 88% for FitHiChIP(S), hichipper and MAPS even though hichipper calls at least four times more loops compared to both FitHiChIP(S) and MAPS **(Fig. 5(a))**. Application of merging filter reduces the number of loop calls as well as the reproducibility between replicates for FitHiChIP, but has a smaller impact on hichipper in both aspects **(Fig. 5(a))**. Similar reproducibility computations for technical replicates of GM12878 cohesin HiChIP data also show that FitHiChIP and MAPS have higher percentage of reproducible loop calls compared to hichipper **(Supplementary Figure 30)**. For K562 H3K27ac HiChIP data results with three replicates 83-93%, 67-84%, 77-85% and 63-87% of the loops (percentages computed with respect to each replicate) are common across all three biological replicates for FitHiChIP(L), FitHiChIP(S), hichipper and MAPS, respectively **(Supplementary Figure 31)**. The relatively lower reproducibility for all methods for cohesin data compared to H3K27ac can be partially attributed to the lower sequencing depth of replicates for the former. However, in general, all three HiChIP loop callers have better reproducibility when compared to similar numbers from existing conformation capture datasets. For instance, with the same overlap criteria we use here, the HiCCUPS loop calls from the two replicates of GM12878 Hi-C data, both sequenced to over two billion reads, have an overlap of 63% (68%) with respect to primary (replicate) sample. For ChIA-PET data with two replicates, these percentages are 50% (59%) and 62% (63%) for GM12878 RAD21 [9] and for K562 Pol2 [15] data, respectively. For comparable number of loop calls, such as hichipper and FitHiChIP(L), reproducibility is generally higher for FitHiChIP. The reduction in reproducibility when merging filter is used correlates with the reduction it causes in the number of loops reported.

In order to further test the robustness of results discovered by FitHiChIP, we analyze mouse embryonic stem cell (mES) HiChIP samples generated from varying number of cells as starting material (cohesin with 1M, 5M and 10M cells, H3K27ac with 50K, 100K, 500K and 25M cells) [19, 20]. Overall, these results show that a significant fraction of the loops called from the sample with lowest cell number (over 95% for cohesin and over 80% for H3K27ac) are also identified in at least one other replicate suggesting that loop calls from low quality samples are still informative. However, in most cases, up to 10 times more loops are identified from the samples with higher cell numbers, which also depends on the sequencing depth, highlighting the importance of library quality and complexity for achieving a comprehensive set of loops from HiChIP **(Supplementary Methods and Supplementary Figure 32)**.

Next, we focus on the overlap between FitHiChIP loop calls from different contact map resolutions for the same data (2.5kb vs 5kb) in order to better understand the impact of window size selection on the resulting loop set. We first perform an overlap analysis to see what fraction of 2.5kb loops are exclusive to this resolution compared to 5kb and vice versa. Our results for three different HiChIP datasets show that, both for loose and stringent background models, over 95% of 2.5kb loops are also detected by an overlapping 5kb loop **(Supplementary Figure 33)**. A substantial fraction of loops detected at 5kb, however, are exclusive to that resolution (41%-72%) potentially due to increased statistical power attained by higher contact density. Among the overlapping set, on average 1.2 to 1.6 number of 2.5kb resolution loops overlap with a 5kb loop call suggesting that multiple pairs among the four possible combinations of 2.5kb bin pairs have significant interactions **(Supplementary Figure 33)**. To further look at the breakdown of such cases, we categorize each 5kb loop that overlaps/contains at least one 2.5kb loop into five main groups. These groups can be summarized as 5kb loops that contain: *(i)* only one 2.5kb loop; two 2.5kb loops *(ii)* with both from one 2.5kb bin on one side or *(iii)*

with loops involving all four bins; *(iv)* three 2.5kb loops and *(v)* four 2.5kb loops **(Fig. 5(b))**. This breakdown demonstrates that either a single underlying 2.5kb loop (group i) or two 2.5kb loops from one single bin on one side (group ii) accounts for 85% to 98% of all 5kb loops that overlap with any 2.5kb call **(Fig. 5(b))**. The unlikely configuration of having two distinct loops with no overlap on either end (group iii) corresponds to less than half a percent as expected **(Fig. 5(b))**. These results suggest that the 2.5kb analysis mainly determines whether the looping information (or the loop anchor) can be resolved to a smaller bin size on either one side or both sides but does not result in discovery of new or distinct loops compared to 5kb analysis.

Supplementary Note 9: Simulating HiChIP experiments by non-uniform sampling of Hi-C contact maps

Unlike Hi-C where each bin/region is expected to have uniform coverage across the genome, HiChIP contact signal is enriched for regions with the targeted histone mark or bound by the transcription factor of interest. In order to understand to what extent an actual HiChIP experiment can be recapitulated by non-uniform sampling of Hi-C contact counts, we generate simulated HiChIP maps using Hi-C and ChIP-seq data such that bins with higher (lower) ChIP-seq signal have higher (lower) "Hi-C" read coverage after the simulation. Such non-uniform sampling cannot be achieved by simply scaling the rows and columns of the Hi-C, which would lead an to asymmetric contact map. Therefore, we adapt the algorithm of scaling strictly positive matrices into specified row and column sums, as described in [16, 25] for matrix balancing to have rows/columns of our simulated maps converge to a desired ChIP-seq coverage vector rather than a uniform number **(Methods)**. This simulation technique also allows us to randomly shuffle the ChIP-seq coverage vector while keeping intact the peak/non-peak designation of each bin to study the impact of ChIP enrichment on HiChIP data.

Using this approach, we create simulated HiChIP contact maps for chromosome 1 from GM12878 *in-situ* Hi-C data [23] as the background and using coverage values from the reference cohesin or H3K27ac ChIP-seq data **(Supplementary Table 1)** to model the relative enrichment. In order to study the relationship between resulting loop calls from simulated HiChIP maps and underlying Hi-C data, we use HiCCUPS loop calls as well as FitHiChIP in peak-to-all mode applied to full set of GM12878 Hi-C data (primary+replicate). We also use FitHiChIP loop calls from the real HiChIP maps as another reference. Using these reference sets, we first ask whether the Hi-C loops are in agreement with the simulated HiChIP data. The simulated cohesin and H3K27ac HiChIP data recovers 72% and 61% of reference HiCCUPS Hi-C loops, respectively, which are comparable to 82% and 87% achieved by the real HiChIP data **(Supplementary Figure 34(a) left panel, Supplementary Figure 35(a) left panel)**. Furthermore, 99% (79%) of loops from the simulated cohesin (H3K27ac) map is supported by the less stringent set of FitHiChIP loop calls from Hi-C data **(Supplementary Figure 34(a) right panel, Supplementary Figure 35(a) right panel)**. These results suggest that FitHiChIP is able to recover the strong loops as well as enriched contacts of the underlying Hi-C data from a simulated HiChIP map. Next, we ask whether the simulation introduces unexpected loops compared to Hi-C and real HiChIP data. Even though the simulation introduces a large number of new loops (so does real HiChIP data) with respect to the very small set of HiCCUPS loops, only 1% (9%) of all loops from simulation are not supported by either of FitHiChIP loops from Hi-C data or from real HiChIP data for cohesin (H3K27ac) **(Supplementary Figure 34(a) right panel, Supplementary Figure 35(a) right panel)**. From the perspective of loops from the real HiChIP data, 35% (26%) of the cohesin (H3K27ac) loops do not overlap with any loops of the simulated data suggesting nearly one third of FitHiChIP loops from HiChIP cannot be explained by either the underlying Hi-C data or by its combination with the non-uniform ChIP-seq coverage **(Supplementary Figure 34(a), Supplementary Figure 35(a))**. The corresponding percentage of loops specific to real HiChIP data are 88% (92%) when compared to the shuffled simulated maps suggesting that the specific enrichment of ChIP-seq coverage in their original locations (such as enhancers, promoters, CTCF binding regions) is critical for simulations that are consistent with real HiChIP experiments **(Supplementary Figure 34(b), Supplementary Figure 35(b))**. Accordingly, shuffled simulations for cohesin (H3K27ac) are only able to capture 12% (14%) of HiCCUPS loops and **(Supplementary Figure 34(b) left panel, Supplementary Figure 35(b) left panel)**. These results suggest that both the statistical power and the recovery of reference loops for FitHiChIP is specific to the case when real HiChIP data is used or when realistic (i.e., not shuffled) HiChIP maps are simulated from Hi-C data

Supplementary Note 10: FitHiChIP is applicable to other conformation capture experiments

To test the applicability of FitHiChIP with minimal amount of modifications on other targeted conformation capture assays, we use GM12878 PCHiC datasets (by combining all replicates) provided in [18]. We use the capture design file corresponding to HindIII RE fragments (provided in [3]) as our reference "peak" set similar to use of ChIP-seq peak files for HiChIP. For comparison of our resulting loop calls, we use CHiCAGO loops with score ≥ 5 for the same dataset as provided in [3].

Overall, FitHiChIP identified a similar number of loops compared to CHiCAGO (∼83k) when we use our stringent background (S:88k, S+M:75k). When we compute loop overlap using 5kb slack, 52% of CHiCAGO loops are covered by FitHiChIP(S) and at least over 88% by loose (L or L+M) background settings, which report 4-6 times higher number of loops **(Supplementary Figure 36(a))**. We then compare FitHiChIP loops on PCHiC data to CHiCAGO loops in terms their performance in recovering HiCCUPS loops computed from either GM12878 *in situ* Hi-C data [23] or GM12878 H3K27ac HiChIP data [20]. In both cases, CHiCAGO loops show marginally higher specificity that is most similar to FitHiChIP(L+M) **(Supplementary Figures 36(e-f))**. FitHiChIP with loose background recovers ∼ 10% more of reference loops but with several times more loop calls than CHiCAGO. We see similar trends using GM12878 RAD21 ChIA-PET loops [9] or FitHiChIP(S+M) calls on GM12878 cohesin and H3K27ac HiChIP datasets as reference **(Supplementary Figures 36(b-d))**. It is important to note that for all reference sets, the maximum recovery achieved by either method on PCHiC data was considerably low (30-40%) compared to recovery plots discussed when we evaluate different methods on HiChIP data. Overall, comparable performance of FitHiChIP to a PCHiC-specific method suggests that our framework is useful for analyzing data from other targeted conformation capture assays.

Supplementary Note 11: Differential loop calling for HiChIP data

Next, we develop a pipeline for identifying differential contact enrichment and loop calling between two different cell lines with replicate HiChIP experiments. Here we use two biological replicates of GM12878 H3K27ac and three replicates of K562 H3K27ac HiChIP data. As HiChIP contact count signal is heavily dependent on the ChIP enrichment of the underlying loci, we utilize matching ChIP-seq tracks (H3K27ac) from ENCODE portal for each cell line **(Supplementary Table 1)** to segregate the contact-level differences into multiple groups with respect to changes in ChIP-seq coverage for both loci involved in a given contact. Analysis of both 1D and 3D changes in finding differential loops is also supported in a recent study [21] which uses Smc1 HiChIP on cancer cell lines, and proposes to find changes in regulatory looping (3D) with or without changes in enhancer (1D) activities. To achieve this grouping with respect to differences in 1D signal, we first classify each 5kb bin in the genome into three categories depending on their ChIP-seq coverage differences between GM12878 and K562 cells. Out of nearly 620k bins, around 25k are classified as having a *high difference* (HD) defined by a statistically significant difference from edgeR [24, 17] applied to underlying ChIP-seq tracks **(Supplementary Figure 37(a), Methods)**. Looping changes involving such loci are likely reflections of the change in "visibility" by HiChIP rather than changes in the underlying 3D conformation. In order to study this, we also define a strict set of bins with *no difference* (ND) as those that are not deemed significant by edgeR and have less than 25% difference in their scaled coverage values between the two cell lines. The remaining bins are labeled as LD for *low difference* **(Supplementary Figure 37(a))**. We first apply edgeR on the union set of all peak-to-all pairs with non-zero contact in at least one of the five replicates, which results in over a million "differential" contacts out of the nearly 20M pairs tested for differences (at an absolute fold change greater than 2 and FDR 5%) **(Supplementary Figure 37(b))**. As expected, contacts that involve a locus with high difference in ChIP-seq coverage (HD) on at least one end have a much higher fraction of differential contacts (25.9% for HD-HD and 13.6% for HD-LD/ND) compared to contacts between two bins with no difference in ChIP-seq (3.6% for ND-ND). Given the very high number of differences many of which may represent changes in the non-significant and likely non-functional portion of chromatin contacts, we further filter them by overlapping the edgeR contact differences with the union set of FitHiChIP loop calls (1% FDR for FitHiChIP(S)) on each replicate of each cell type **(Supplementary Figure 37(b))**. This resulted in an increased stringency and an overall reduction of more than 15 fold (∼1.67M to ∼106k) leaving only 1,131 (out of 17,466) for ND-ND and 18,841 loops (out of 91,697) for HD-HD. Our reasoning for focusing mainly on this subset with differential loops, rather than other differential contact enrichments without any loop, is that differences restricted to the null portion (not rejected by FitHiChIP's test) of HiChIP data in both cell types are less likely to have functional consequences.

In order to characterize the strength of support for various categories of the differential loops discussed above, we further analyze the underlying HiChIP data, ChIP-seq data as well as the scaled *in situ* Hi-C contact counts for GM12878 and K562 **(Methods)**. First we ask whether the loops in the FitHiChIP overlapping portion of the ND-ND subset (between two bins with no underlying ChIP-seq change) of edgeR differential calls (ND-ND edgeR+FitHiChIP) have stronger local enrichment patterns compared to those from the overall subset (ND-ND edgeR). APA plots made from underlying HiChIP data for all ND-ND differential calls show an enrichment with respect to local background for the cell type with the higher contact count (APA scores of 2.32 and 3.32) but not for the other (APA scores of 1.2 and 1.26) **(Supplementary Figure 37(c) - top row)**. When we repeat the same analysis for the ND-ND edgeR+FitHiChIP set **(Supplementary Figure 37(d) - top row)**, the gap between APA scores for a given subset (e.g., differential calls with loops exclusive to GM12878) between the two cell types increases markedly (4.93 and 5.96 versus 1.83 and 2.35). This suggests that differential calls overlapping with FitHiChIP for ND-ND correspond to regions with enriched contact signals in both cell types but that enrichment is substantially stronger for the cell type with a significant FitHiChIP loop **(Supplementary**

Figure 37(d) - top row). On the other hand, contacts for locus pairs from differential calls with high difference of ChIP-seq signal on both ends that overlap with FitHiChIP loops (HD-HD edgeR+FitHiChIP) have very strong local enrichment in one cell type (APA of 6.17 and 4.41), which is completely absent from the other (APA of 0.65 and 1.03). This is expected as HD-HD differences mainly reflect loops related to cell type-specific ChIP-seq peaks **(Supplementary Figure 37(e) - top row)**. This is also evident from the plots showing the distribution of percent difference in ChIP-seq coverage values **(Supplementary Figure 37(c-e) - middle row)** for all three cases (ND-ND edgeR, ND-ND edgeR+FitHiChIP, HD-HD edgeR+FitHiChIP).

As the last part of our analysis on these three different sets, we use GM12878 and K562 Hi-C datasets [23] to compute a log fold change (K562/GM12878) between the scaled Hi-C contact counts corresponding to each differential call **(Methods)**. We observe some agreement between HiChIP differential calls and the direction of underlying changes in Hi-C counts for each of the three different sets analyzed **(Supplementary Figure 37(c-e) - bottom row)**. However, the relationship is far from complete and a substantial portion of Hi-C fold changes go in the opposite direction suggesting some of the differences seen in HiChIP are not fully explained by either the ChIP-seq signal (for ND-ND case) or the underlying Hi-C data. Another important observation was that when 1D categories such ND, LD and HD are determined using HiChIP 1D coverages instead of that from ChIP-seq, we see a very minimal number of differential loops in the group ND-ND, suggesting that differential loops also involve differential HiChIP coverage values. Overall, these analyses highlight the importance of refining differential HiChIP analysis using FitHiChIP loop calls to get a subset of more pronounced differences, which are also more likely to have functional implications as they specifically link distal regulatory elements (H3K27ac in this case) in at least one cell type.

Supplementary References

[1] Ferhat Ay, Timothy L. Bailey, and William S. Noble. Statistical confidence estimation for Hi-C data reveals regulatory chromatin contacts. *Genome Research*, 24(6):999–1011, 2014.

[2] Ferhat Ay and William S. Noble. Analysis methods for studying the 3D architecture of the genome. *Genome Biology*, 16(1):183, Sep 2015.

[3] J. Cairns, P. Freire-Pritchett, S. W. Wingett, C. Varnai, A. Dimond, V. Plagnol, D. Zerbino, S. Schoenfelder, B. M. Javierre, C. Osborne, P. Fraser, and M. Spivakov. CHiCAGO: robust detection of DNA looping interactions in Capture Hi-C data. *Genome Biol.*, 17(1):127, Jun 2016.

[4] F. Cao, Y. Fang, H. K. Tan, Y. Goh, J. Y. H. Choy, B. T. H. Koh, J. Hao Tan, N. Bertin, A. Ramadass, E. Hunter, J. Green, M. Salter, A. Akoulitchev, W. Wang, W. J. Chng, D. G. Tenen, and M. J. Fullwood. Super-Enhancers and Broad H3K4me3 Domains Form Complex Gene Regulatory Circuits Involving Chromatin Interactions. *Sci Rep*, 7(1):2186, May 2017.

[5] J. M. Dowen, Z. P. Fan, D. Hnisz, G. Ren, B. J. Abraham, L. N. Zhang, A. S. Weintraub, J. Schujiers, T. I. Lee, K. Zhao, and R. A. Young. Control of cell identity genes occurs in insulated neighborhoods in mammalian chromosomes. *Cell*, 159(2):374–387, Oct 2014.

[6] R. Fang, M. Yu, G. Li, S. Chee, T. Liu, A. D. Schmitt, and B. Ren. Mapping of long-range chromatin interactions by proximity ligation-assisted ChIP-seq. *Cell Res.*, 26(12):1345–1348, Dec 2016.

[7] C. P. Fulco, M. Munschauer, R. Anyoha, G. Munson, S. R. Grossman, E. M. Perez, M. Kane, B. Cleary, E. S. Lander, and J. M. Engreitz. Systematic mapping of functional enhancer-promoter connections with CRISPR interference. *Science*, 354(6313):769–773, 11 2016.

[8] M. Gasperini, A. Hill, J. L. McFaline-Figueroa, B. Martin, S. Kim, M. D. Zhang, D. Jackson, A. Leith, J. Schreiber, W. S. Noble, C. Trapnell, N. Ahituv, and J. Shendure. A genome-wide framework for mapping gene regulation via cellular genetic screens. *Cell*, 176:1–14, Jan 2019.

[9] N. Heidari, D. H. Phanstiel, C. He, F. Grubert, F. Jahanbani, M. Kasowski, M. Q. Zhang, and M. P. Snyder. Genome-wide map of regulatory interactions in the human genome. *Genome Res.*, 24(12):1905–1917, Dec 2014.

[10] J. Huang, K. Li, W. Cai, X. Liu, Y. Zhang, S. H. Orkin, J. Xu, and G. C. Yuan. Dissecting super-enhancer hierarchy based on chromatin interactions. *Nat Commun*, 9(1):943, 03 2018.

[11] M. Imakaev, G. Fudenberg, R. P. McCord, N. Naumova, A. Goloborodko, B. R. Lajoie, J. Dekker, and L. A. Mirny. Iterative correction of Hi-C data reveals hallmarks of chromosome organization. *Nat. Methods*, 9(10):999–1003, Oct 2012.

[12] B. M. Javierre, O. S. Burren, S. P. Wilder, R. Kreuzhuber, S. M. Hill, S. Sewitz, J. Cairns, S. W. Wingett, C. Varnai, M. J. Thiecke, F. Burden, S. Farrow, A. J. Cutler, K. Rehnstrom, K. Downes, L. Grassi, M. Kostadima, P. Freire-Pritchett, F. Wang, H. G. Stunnenberg, J. A. Todd, D. R. Zerbino, O. Stegle, W. H. Ouwehand, M. Frontini, C. Wallace, M. Spivakov, P. Fraser, J. H. Martens, B. Kim, N. Sharifi, E. M. Janssen-Megens, M. L. Yaspo, M. Linser, A. Kovacsovics, L. Clarke, D. Richardson, A. Datta, and P. Flicek. Lineage-Specific Genome Architecture Links Enhancers and Non-coding Disease Variants to Target Gene Promoters. *Cell*, 167(5):1369–1384, 11 2016.

[13] I. Juric, M. Yu, A. Abnousi, R. Raviram, R. Fang, Y. Zhao, Y. Zhang, Y. Qiu, Y. Yang, Y. Li, B. Ren, and M. Hu. MAPS: Model-based analysis of long-range chromatin interactions from PLAC-seq and HiChIP experiments. *PLoS Comput. Biol.*, 15(4):e1006982, 04 2019.

[14] C. A. Lareau and M. J. Aryee. hichipper: a preprocessing pipeline for calling DNA loops from HiChIP data. *Nat. Methods*, 15(3):155–156, Feb 2018.

[15] G. Li, X. Ruan, R. K. Auerbach, K. S. Sandhu, M. Zheng, P. Wang, H. M. Poh, Y. Goh, J. Lim, J. Zhang, H. S. Sim, S. Q. Peh, F. H. Mulawadi, C. T. Ong, Y. L. Orlov, S. Hong, Z. Zhang, S. Landt, D. Raha, G. Euskirchen, C. L. Wei, W. Ge, H. Wang, C. Davis, K. I. Fisher-Aylor, A. Mortazavi, M. Gerstein, T. Gingeras, B. Wold, Y. Sun, M. J. Fullwood, E. Cheung, E. Liu, W. K. Sung, M. Snyder, and Y. Ruan. Extensive promoter-centered chromatin interactions provide a topological basis for transcription regulation. *Cell*, 148(1-2):84–98, Jan 2012.

[16] Albert W. Marshall and Ingram Olkin. Scaling of matrices to achieve specified row and column sums. *Numerische Mathematik*, 12(1):83–90, Aug 1968.

[17] D. J. McCarthy, Y. Chen, and G. K. Smyth. Differential expression analysis of multifactor RNA-Seq experiments with respect to biological variation. *Nucleic Acids Res.*, 40(10):4288–4297, May 2012.

[18] B. Mifsud, F. Tavares-Cadete, A. N. Young, R. Sugar, S. Schoenfelder, L. Ferreira, S. W. Wingett, S. Andrews, W. Grey, P. A. Ewels, B. Herman, S. Happe, A. Higgs, E. LeProust, G. A. Follows, P. Fraser, N. M. Luscombe, and C. S. Osborne. Mapping long-range promoter contacts in human cells with high-resolution capture Hi-C. *Nat. Genet.*, 47(6):598–606, Jun 2015.

[19] M. R. Mumbach, A. J. Rubin, R. A. Flynn, C. Dai, P. A. Khavari, W. J. Greenleaf, and H. Y. Chang. HiChIP: efficient and sensitive analysis of protein-directed genome architecture. *Nat. Methods*, 13(11):919–922, Nov 2016.

[20] M. R. Mumbach, A. T. Satpathy, E. A. Boyle, C. Dai, B. G. Gowen, S. W. Cho, M. L. Nguyen, A. J. Rubin, J. M. Granja, K. R. Kazane, Y. Wei, T. Nguyen, P. G. Greenside, M. R. Corces, J. Tycko, D. R. Simeonov, N. Suliman, R. Li, J. Xu, R. A. Flynn, A. Kundaje, P. A. Khavari, A. Marson, J. E. Corn, T. Quertermous, W. J. Greenleaf, and H. Y. Chang. Enhancer connectome in primary human cells identifies target genes of disease-associated DNA elements. *Nat. Genet.*, 49(11):1602–1612, Nov 2017.

[21] J. Petrovic, Y. Zhou, M. Fasolino, N. Goldman, G. W. Schwartz, M. R. Mumbach, S. C. Nguyen, K. S. Rome, Y. Sela, Z. Zapataro, S. C. Blacklow, M. J. Kruhlak, J. Shi, J. C. Aster, E. F. Joyce, S. C. Little, G. Vahedi, W. S. Pear, and R. B. Faryabi. Oncogenic Notch Promotes Long-Range Regulatory Interactions within Hyperconnected 3D Cliques. *Mol. Cell*, 73(6):1174–1190, 03 2019.

[22] D. H. Phanstiel, A. P. Boyle, N. Heidari, and M. P. Snyder. Mango: a bias-correcting ChIA-PET analysis pipeline. *Bioinformatics*, 31(19):3092–3098, Oct 2015.

[23] S. S. Rao, M. H. Huntley, N. C. Durand, E. K. Stamenova, I. D. Bochkov, J. T. Robinson, A. L. Sanborn, I. Machol, A. D. Omer, E. S. Lander, and E. L. Aiden. A 3D map of the human genome at kilobase resolution reveals principles of chromatin looping. *Cell*, 159(7):1665–1680, Dec 2014.

[24] M. D. Robinson, D. J. McCarthy, and G. K. Smyth. edgeR: a Bioconductor package for differential expression analysis of digital gene expression data. *Bioinformatics*, 26(1):139–140, Jan 2010.

[25] Uriel G. Rothblum and Hans Schneider. Scalings of matrices which have prespecified row sums and column sums via optimization. *Linear Algebra and its Applications*, 114-115:737 – 764, 1989. Special Issue Dedicated to Alan J. Hoffman.

[26] Y. Zhang, T. Liu, C. A. Meyer, J. Eeckhoute, D. S. Johnson, B. E. Bernstein, C. Nusbaum, R. M. Myers, M. Brown, W. Li, and X. S. Liu. Model-based analysis of ChIP-Seq (MACS). *Genome Biol.*, 9(9):R137, 2008.
